# (*aS*)-Glucosciadopitysin, a New Biflavonoid Glycoside from the Leaves of *Ginkgo biloba* and Osteogenic Activity of Bioflavonoids

**DOI:** 10.3390/plants14020261

**Published:** 2025-01-17

**Authors:** Se Yun Jeong, Kwang Ho Lee, Seon Hee Kim, Min Hye Yang, Gakyung Lee, Ki Hyun Kim

**Affiliations:** 1 School of Pharmacy, Sungkyunkwan University, Suwon 16419, Republic of Korea; dlawktkark@naver.com (S.Y.J.); sholaly@naver.com (K.H.L.); 2 Research Institute, Sungkyun Biotech Co., Ltd., Anyang 14118, Republic of Korea; seonhee31@gmail.com; 3 Department of Pharmacy, College of Pharmacy and Research Institute for Drug Development, Pusan National University, Busan 46241, Republic of Korea; 4 Department of Integrative Biological Sciences and Industry, Sejong University, Seoul 05006, Republic of Korea

**Keywords:** *Ginkgo biloba*, NMR analysis, electronic circular dichroism (ECD) calculations, mesenchymal stem cell, alkaline phosphatase

## Abstract

The leaves of *Ginkgo biloba* have been used in treating freckles and effectively reducing cough and sputum in folk medicines. Recently, investigations into the correlation between ginkgo leaves and the proliferative activity of osteogenic differentiation have been conducted. However, bioactive compounds that enhance osteogenesis or exhibit osteoporosis prevention from *G. biloba* have not been fully identified. Phytochemical investigation of the MeOH extract of *G. biloba* leaves led to the isolation and identification of a new biflavonoid glycoside, (*aS*)-glucosciadopitysin (**1**), along with five flavonoids (**2**–**6**), through LC/MS-guided isolation approach. The structure of the new compound **1** was elucidated by the spectroscopic methods, including 1D and 2D NMR analysis, as well as HR-ESIMS. The absolute configuration of sugar moiety was established through acid hydrolysis, followed by chemical derivatization reaction and the axial chirality arising from the biaryl system with substituents was determined by electronic circular dichroism (ECD) calculations. The isolated flavonoids (**1**–**6**) were tested for their effects on mesenchymal stem cell (MSC) differentiation at 20 μM using Oil Red O and alkaline phosphatase (ALP) staining. Ginkgetin (**2**) was further evaluated for osteogenic activity on C3H10T1/2 cells at concentrations of 1, 2.5, 5, and 10 μM for 10 days. ALP staining and RT-PCR assessed the gene expression of osteogenic markers ALP and osteopontin (OPN). Ginkgetin (**2**) demonstrated the strongest osteogenic activity, significantly increasing the expression of ALP (12.5-fold) and OPN (4.0-fold) at 10 μM, comparable to the positive control, oryzativol A. Ginkgetin (**2**) shows potential as a therapeutic agent for osteopenia by promoting osteogenesis in MSCs, suggesting its promising role in treating osteoporosis.

## 1. Introduction

Mesenchymal stem cells (MSCs) are adult stem cells found in the bone marrow. They possess the unique ability to proliferate in an undifferentiated state and can differentiate into various mesenchymal cell types, including bone cells, chondrocytes, and adipocytes [1,2]. The expression of alkaline phosphatase (ALP) and osteopontin (OPN) serves as early and prominent markers of osteogenic differentiation in MSCs [3,4]. ALP, a ubiquitous ectoenzyme, plays a crucial role in maintaining MSC stemness and preventing bone ageing by regulating ATP-mediated AMPKα alterations [5,6]. OPN, on the other hand, is a highly phosphorylated sialoprotein with a high calcium-binding affinity. It is an essential component of the mineralized extracellular matrix, contributing to the construction of bones and teeth [7]. These markers are integral to understanding the osteogenic differentiation process and hold significance in research aimed at elucidating mechanisms underlying bone formation and regeneration.

*Ginkgo biloba* L. (Ginkgoaceae) is extensively grown as a decorative tree in urban areas and parks. *G*. *biloba*, commonly known as ginkgo, is a species of gymnosperm tree native to East Asia, and it is widely utilized as a medicinal plant, notably in countries such as Japan, China, and Korea [8]. Traditionally, the leaves of *G. biloba* have demonstrated medicinal efficacy and have been used to treat various illnesses such as freckles, cough, and sputum, according to ancient Chinese literature [9]. Research on *G. biloba* leaves has identified diverse bioactive compounds, including terpene trilactones (ginkgolides), alkylphenols (ginkgolic acids), and flavonoids, such as biflavonoids (ginkgetin) [10]. Recent studies have further explored the relationship between these compounds and their bioactivity. For example, ginkgolic acid derivatives, which inhibit protein tyrosine phosphatases (PTPs), are considered potential therapeutic candidates for combating type 2 diabetes mellitus [11]. Additionally, biflavonoids such as bilobetin, ginkgetin, and isoginkgetin have shown potential benefits in managing metabolic syndrome, including obesity and diabetes, by regulating several enzymes/proteins and insulin signalling pathways [12].

Recently, investigations that focus on the correlation between ginkgo leaves and the proliferative activity of osteogenic differentiation have been conducted [13,14]. The ethanolic extract of *G. biloba* has been shown to improve the proliferation and osteogenesis of human bone marrow-derived MSCs in a dose-dependent manner, as indicated by ALP activity and calcium content [13]. Furthermore, *G. biloba* extract inhibited adipocyte differentiation and enhanced osteogenic differentiation in bone marrow-derived MSCs by overexpression of osteogenic-related genes such as BMP-2, Runx2, and Colla1 [14]. Meanwhile, *G. biloba* extract exhibited anti-osteoporotic effects in glucocorticoid-induced osteoporosis and significantly inhibited the loss of alveolar bone in the mandible [15]. Recent study has demonstrated that ginkgolide B, a constituent of *G. biloba* leaves, promotes osteoblast differentiation and bone formation through the activation of canonical Wnt signalling pathways. Additionally, it has been shown to alleviate ovariectomy-induced osteoporosis by enhancing osteoblast activity [16]. However, the exploration of bioactive compounds that enhance the osteogenesis of MSCs or exhibit osteoporosis prevention from *G. biloba* has been limited.

As part of our ongoing investigation in search of structurally novel and bioactive compounds from intriguing natural resources [17,18,19,20,21], we investigated a methanol (MeOH) extract of *G. biloba* leaves. Liquid chromatography–mass spectrometry (LC/MS) analysis of fractions derived from the MeOH extract facilitated the isolation process, resulting in the separation of a new biflavonoid glycoside (**1**) along with five flavonoids (**2**–**6**). The chemical structure of the new compound (**1**) was elucidated using HR-ESIMS, 1D and 2D NMR (COSY, HSQC, and HMBC) analysis. The identification of sugar moiety was determined through chemical transformation, followed by acid hydrolysis. Subsequently, the absolute configuration of compound **1** as an atropisomer was deduced by comparing experimental and calculated electronic circular dichroism (ECD) spectra. Here, we present the isolation and structural characterization of compounds **1**–**6** along with an evaluation of their regulatory effects on mesenchymal stem cell (MSC) differentiation into adipocytes and osteoblasts.

## 2. Results and Discussion

### 2.1. Isolation of Compounds from the Leaves of G. biloba

**Figure 1:**
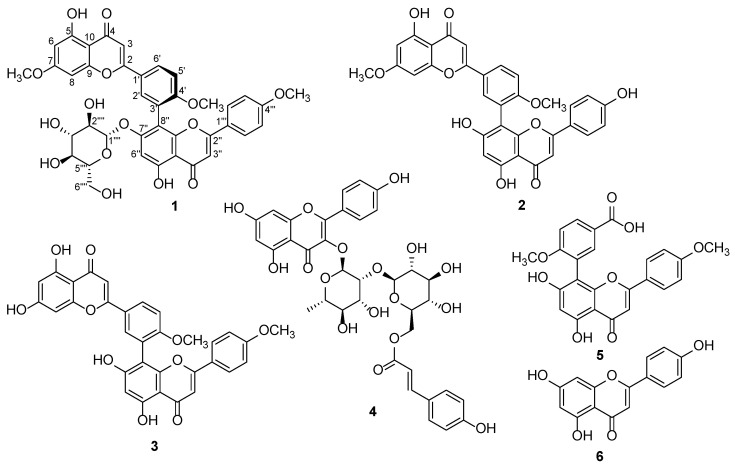
Chemical structures of compounds **1**–**6**.

The dried leaves of *G. biloba* were extracted with 80% aqueous MeOH to obtain a crude MeOH extract. The extract was then solvent-partitioned with four organic solvents to give four main fractions, among which the hexane-soluble fraction was subjected to analysis using column chromatography techniques. Medium-pressure liquid chromatography (MPLC) and high-performance liquid chromatography (HPLC) were conducted for the phytochemical investigation of the hexane-soluble fraction, with LC/MS analysis combined with our in-house UV library database. The phytochemical investigations resulted in the isolation of flavonoids (**1**–**6**) (Figure 1), including a new biflavonoid glycoside termed (*aS*)-glucosciadopitysin (**1**).

Compound **1** was isolated as a yellowish plate, and the HR-ESIMS data of **1** demonstrated the protonated molecular ion peak [M + H]^+^ at *m/z* 743.1977 (calculated for C_39_H_35_O_15_^+^, 743.1970) in the positive-ion mode, which was compatible with the molecular formula of C_39_H_34_O_15_ (Figure S1). Detailed interpretation of the ^1^H NMR data (Figure S3), combined with HSQC (Figure S6 and Table 1) revealed the presence of two sets of flavonoid skeleton at H-6/C-6 [*δ*_H_ 6.35 (1H, d, *J* = 2.0 Hz)/*δ*_C_ 97.9], H-8/C-8 [*δ*_H_ 6.66 (1H, d, *J* = 2.0 Hz)/*δ*_C_ 91.9], H-3/C-3 [*δ*_H_ 6.79 (1H, s)/*δ*_C_ 103.4], H-5′/C-5′ [*δ*_H_ 7.35 (1H, d, *J* = 8.5 Hz)/*δ*_C_ 111.1], H-2′/C-2′ [*δ*_H_ 8.08 (1H, d, *J* = 2.0 Hz)/*δ*_C_ 131.2] and H-6′/C-6′ [*δ*_H_ 8.16 (1H, dd, *J* = 8.5, 2.0 Hz)/*δ*_C_ 128.1], as well as H-3′′/C-3′′ [*δ*_H_ 6.75 (1H, s)/*δ*_C_ 102.6], H-6′′/C-6′′ [*δ*_H_ 6.82 (1H, s)/*δ*_C_ 98.5], H-3′′′,H-5′′′/C-3′′′,C-5′′′ [*δ*_H_ 6.92 (2H, d, *J* = 9.0 Hz)/*δ*_C_ 114.1], and H-2′′′,H-6′′′/C-2′′′,C-5′′′ [*δ*_H_ 7.59 (2H, d, *J* = 9.0 Hz)/*δ*_C_ 127.6]. Three sets of the methoxy group were also assigned at 7-OCH_3_ [*δ*_H_ 3.87 (3H, s)/*δ*_C_ 55.0], 4′-OCH_3_ [*δ*_H_ 3.81 (3H, s)/*δ*_C_ 54.9] and 4′′′-OCH_3_ [*δ*_H_ 3.80 (3H, s)/*δ*_C_ 54.5]. Peaks within the *δ*_H_ 3.25–3.92 range, along with an anomeric proton resonating at H-1⁗ [*δ*_H_ 5.19 (1H, d, *J* = 7.5 Hz)] and a corresponding carbon signal at *δ*_C_ 100.1, were assigned as part of a sugar moiety, which was confirmed through ^1^H-^1^H COSY correlation (Figure S4), revealing correlations of H-1⁗/H-2⁗/H-3⁗/H-4′′′′/H-5⁗/H-6⁗. The chemical shifts in the corresponding carbon signals for the sugar moiety and the coupling constant (*J* = 7.5 Hz) of the anomeric proton at *δ*_H_ 5.19 matched well with previously reported data [22], confirming the sugar moiety as *β*-glucopyranose.

**Figure 2:**
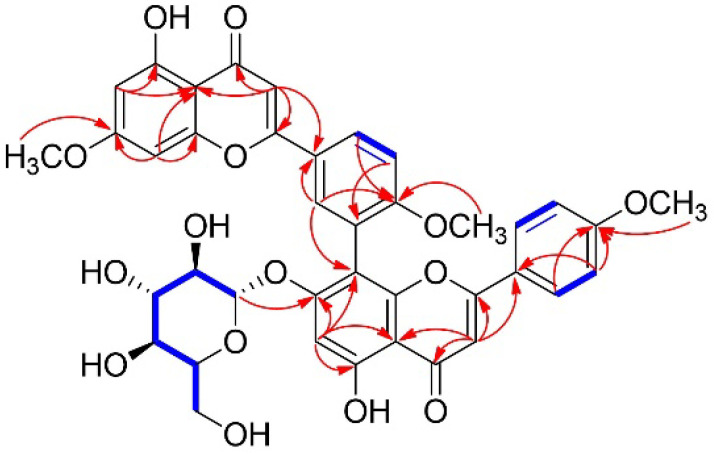
Key ^1^H-^1^H COSY (blue bold) and HMBC (red arrow) correlations for **1**.

The planar structure of **1** was fully elucidated with the assistance of ^1^H-^1^H COSY and HMBC analysis (Figure 2 and Figure S7). The following correlations helped to confirm the presence of one flavonoid moiety: ^1^H-^1^H COSY correlation of H-5′/H-6′, and HMBC correlations of H-3/C-2, C-4, C-10, and C-1′; H-6/C-5, C-8, and C-10; H-8/C-6, C-7, C-9, and C-10; H-2′/C-1′, C-4′, and C-6′; H-5′/C-1′ and C-3′; and H-6′/C-2′ and C-4′. Furthermore, the following correlations allowed us to confirm the presence of another flavonoid moiety: ^1^H-^1^H COSY correlation of H-2′′′/H-3′′′, and HMBC correlations of H-3′′/C-2′′, C-4′′, C-10′′, and C-1′′′; H-6′′/C-5′′, C-7′′, C-8′′, and C-10′′; H-2′′′/C-4′′′ and C-6′′′; and H-3′′′/C-1′′′, C-4′′′, and C-5′′′. The linkage of two flavonoid substructures was identified between C-3′ and C-8′′, which was verified by the HMBC correlation of H-2′/C-8′′. The positions of the three methoxy groups were confirmed by HMBC correlations: 7-OCH_3_ (*δ*_H_ 3.87)/C-7; 4′-OCH_3_ (*δ*_H_ 3.81)/C-4′; and 4′′′-OCH_3_ (*δ*_H_ 3.80)/C-4′′′. The carbon linked to the sugar moiety was confirmed at C-7′′ via the HMBC correlation of H-1⁗/C-7′′. Consequently, the complete planar structure of **1** was determined as shown in Figure 1.

To assign the absolute configuration of sugar moiety for compound **1**, acid hydrolysis followed by a chemical derivatization reaction was performed to determine the D/L configuration of *β*-glucopyranose (Figure S8) [24]. *β*-Glucopyranose obtained from the acid hydrolysis of compound **1** and standard of *β*-D-glucopyranose underwent a chemical reaction to obtain thiocarbamoyl-thiazolidine derivatives. Their retention times were compared using LC/MS analysis, which revealed that *β*-glucopyranose from compound **1** was identified as D-form, as they showed the same retention time (*t*_R_ = 19.5 min).

**Figure 3:**
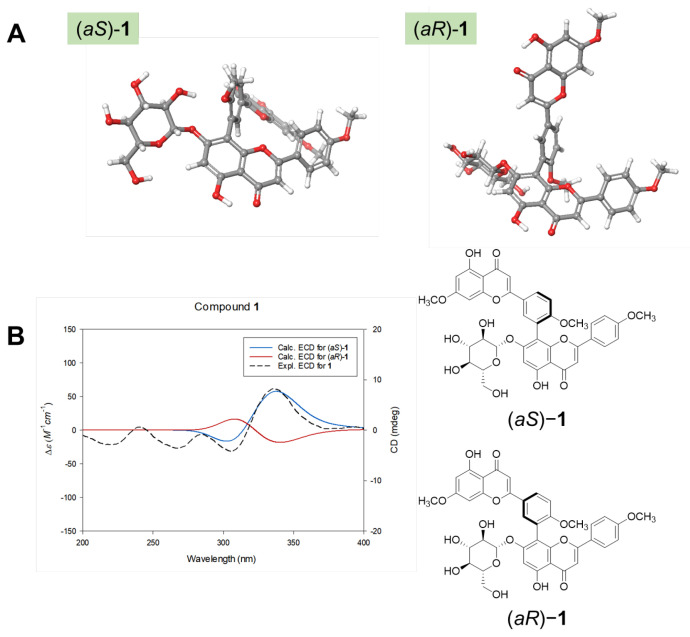
(**A**) Possible three-dimensional structures of two isomers, (*aS*)-**1** and (*aR*)-**1**, and (**B**) experimental and calculated ECD spectra for **1**.

Compound **1** possesses flavone residues linked at 3′ and 8′′, forming a heterodimeric biaryl system. The steric hindrance of 4′-OCH_3_ and 7′′-OGlc in the biaryl system results in a high rotational energy barrier for compound **1**, leading to atropisomerism [25]. Practically, two biflavonoid glycosides with different axial chirality from *Malus hupehensis* Pamp. (Rosaceae) exhibited opposite cotton effects [26]. To determine the absolute configuration and axial chirality of compound **1**, quantum chemical calculations for ECD simulations were performed. ECD data were simulated for two isomers, (*aS*)-**1** and (*aR*)-**1**, and the calculated ECD data were compared with the experimental ECD spectrum of **1** (Figure 3). As a result, the experimental ECD data matched with the calculated ECD data of (*aS*)-**1**, indicating that the absolute configuration and axial chirality of compound **1** were definitively determined to be (*aS*). Accordingly, the chemical structure of compound **1**, including the absolute configuration and axial chirality, was determined (Figure 3) and designated as (*aS*)-glucosciadopitysin.

The other isolated compounds were identified as ginkgetin (**2**) [27], isoginkgetin (**3**) [27], kaempferol 3-*O*-*β*-D-(6′′-*p*-coumaroyl)glucopyranosyl(1→2)-*α*-L-rhamnopyranoside (**4**) [28], 3-[5,7-dihydroxy-2-(4-methoxyphenyl)-4-oxo-4*H*-1-benzopyran-8-yl]-4-methoxybenzoic acid (**5**) [29] and apigenin (**6**) [30] through a comparison of their NMR spectra with the reported data and MS data from their LC/MS analysis (Figures S9-S18, Tables S1 and S2).

### 2.2. The Regulatory Effects of Compounds **1**–**6** on MSC Differentiation into Adipocytes and Osteoblasts

**Figure 4:**
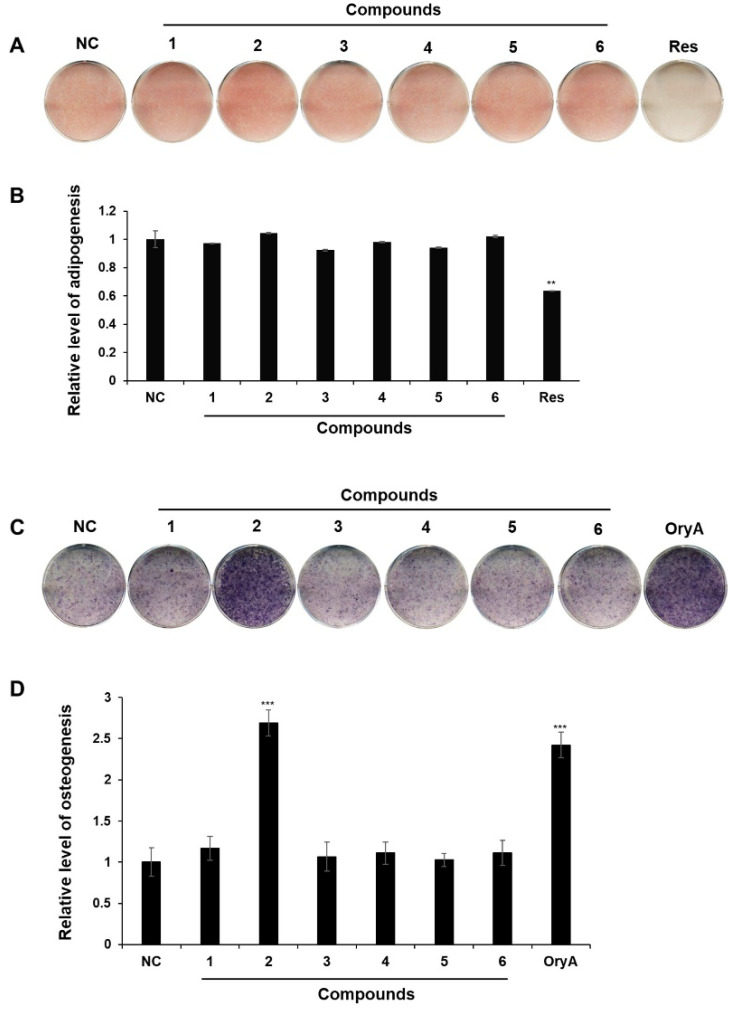
The effects of isolated compounds **1**–**6** on MSC differentiation into osteoblasts or adipocytes using the mouse MSC line, C3H10T1/2. Adipogenic differentiation was assessed by staining cells with Oil Red O (ORO) (**A**) and quantifying stained lipid droplets’ absorbance at a red stain wavelength (**B**). Osteoblast differentiation was evaluated through alkaline phosphatase (ALP) staining (**C**), with the intensity of stained cells measured (**D**). The untreated negative control is denoted as NC. Adipogenesis was induced using a 20 μM concentration of resveratrol (Res) as a positive control, while osteogenesis was stimulated using 5 μM oryzativol A (OryA). Each compound was applied at a concentration of 20 μM in adipogenesis- or osteogenesis-inducing media. Statistical significance: ** *p* < 0.01; *** *p* < 0.005.

To assess the regulatory effects of the isolated compounds **1**–**6** on MSC differentiation into adipocytes and osteoblasts, the mouse mesenchymal stem cell line C3H10T1/2 was exposed to a concentration of 20 µM of compounds **1**–**6** during adipogenic or osteogenic differentiation. While the six isolated compounds showed no significant impact on the adipocyte differentiation process of MSCs, compound **2** exhibited a notable enhancement of osteogenic differentiation at a concentration of 20 μM, surpassing the effects observed with the other isolated compounds (Figure 4).

**Figure 5:**
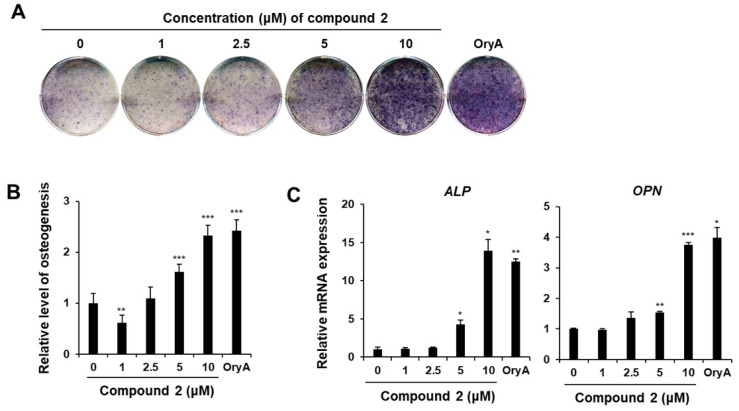
The stimulatory effects of compound **2** on osteogenic differentiation. The mouse MSC line, C3H10T1/2, was treated with compound **2** in osteogenic differentiation media. ALP staining was conducted to assess osteoblast formation, with untreated cells marked as 0. Fully differentiated cells were stained with ALP at 10 days post-osteogenic differentiation with varying concentrations of compound **2** (**A**). ALP activity was measured in osteogenic-differentiated MSCs treated with different concentrations of compound **2** (**B**). Gene expression levels of osteogenic markers, *ALP* and *OPN*, were evaluated (**C**). Oryzativol A (5 μM) was used as a positive control. Statistical significance: * *p* ≤ 0.05; ** *p* < 0.01; *** *p* < 0.001.

When compound **2** was introduced into the culture medium of MSCs undergoing osteogenic differentiation, ranging from 1 to 10 μM concentrations, the intensity of alkaline phosphatase (ALP) staining increased proportionally with the escalating concentration of compound **2**, comparable to the active positive control, oryzativol A (Figure 5). This observation suggests that the process of osteogenic differentiation is facilitated in a dose-dependent fashion by compound **2**. MSCs cultured in an osteogenic differentiation medium containing compound **2** exhibited a noticeable increase in the expression of osteopontin (*OPN*) and alkaline phosphatase (*ALP*), markers typically associated with bone and tooth formation (Figure 5). Osteogenic differentiation of MSCs was induced in the osteogenic differentiation medium, followed by mRNA analysis on the 10th day of culture. This analysis revealed a proportional increase in the expression levels of osteogenic markers *ALP* and *OPN* in response to varying concentrations of compound **2**. At the highest concentration of 10 μM, the gene expression of *ALP* and *OPN* was, respectively, 13.9-fold and 3.8-fold higher compared to the untreated negative control group, which is comparable to 12.5-fold and 4.0-fold in the positive control group, oryzativol A.

We discovered that ginkgetin (**2**) shows promise for promoting osteogenic differentiation. Our findings suggest that the position of the methoxy group can be critical for this effect. Specifically, the presence of the methoxy group at the C-7 position appears to play a significant role in osteogenic differentiation, as evidenced by the comparison of ginkgetin (**2**) and isoginkgetin (**3**). Similarly, previous reports on structure–activity relationships have suggested that the number and position of methoxy groups in biflavonoids can influence polarity modification and the ability to establish hydrogen bonds with target proteins [31]. Additionally, we observed that the functional group attached at C-7′′ is also able to affect osteogenic differentiation activity, as demonstrated in the comparison of compounds **1** and **2**. This insight provides a rationale for the potential usefulness of *G. biloba* extract in promoting osteogenic differentiation, given that ginkgetin (**2**) is a prominent component of *G. biloba* [32].

## 3. Materials and Methods

### 3.1. General Experimental Procedures

Optical rotations were measured using a Jasco P-2000 polarimeter (Jasco, Easton, MD, USA). Ultraviolet (UV) spectra were acquired on an Agilent 8453 UV-visible spectrophotometer (Agilent Technologies, Santa Clara, CA, USA). Electronic circular dichroism spectra were measured on a Jasco J-1500 spectropolarimeter (Jasco). Infrared (IR) spectra were recorded with a Bruker VERTEX 70 FT-IR spectrometer (Bruker, Karlsruhe, Germany). Nuclear magnetic resonance (NMR) spectra were recorded with a Bruker AVANCE III HD 850 NMR spectrometer with a 5 mm TCI CryoProbe operating at 850 MHz (^1^H) and 212.5 MHz (^13^C) (Bruker, Karlsruhe, Germany), with chemical shifts given in ppm (*δ*) for ^1^H and ^13^C NMR analyses, and they were referenced to solvent peaks of CD_3_OD at 3.310 ppm for ^1^H and 49.000 ppm for ^13^C (Cambridge Isotope Laboratories, Tewksbury, MA, USA). The HR-ESI-MS data were obtained with an Agilent 6545 Q-TOF LC/MS spectrometer using an EclipsePlus C18 95 Å column (50 × 2.1 mm, 1.8 μm; flow rate: 0.3 mL/min; Agilent Technologies, Santa Clara, CA, USA). LC/MS analysis was performed on an Agilent 1200 Series HPLC system equipped with a diode array detector and 6130 Series ESI mass spectrometer using an analytical Kinetex C18 100 Å column (100 × 2.1 mm, 5 μm; flow rate: 0.3 mL/min; Phenomenex, Torrance, CA, USA). MPLC was carried out using a Yamazen Smart Flash AKROS system (Yamazen Corporation, Osaka, Japan) with an ODS-SM column (16.5 × 3.0 cm, 50 μm; flow rate: 20 mL/min; Yamazen Corporation) for reversed-phase MPLC. HPLC was performed using a Waters 1525 Binary HPLC pump with a Waters 996 Photodiode Array Detector (Waters Corporation, Milford, CT, USA) and a HECTOR C18 column (250 × 21.2 mm, 5 μm; flow rate: 5 mL/min; RStech, Chungcheongbuk-do, Cheong-Ju, Republic of Korea). Semi-preparative HPLC was performed using a Shimadzu Prominence HPLC System with SPD-20A/20AV Series Prominence HPLC UV-Vis detectors (Shimadzu, Tokyo, Japan) with a Phenomenex Luna Phenyl-Hexyl column (250 × 10 mm, 10 μm; flow rate: 2 mL/min; Phenomenex). Silica gel 60 (230-400 mesh; Merck, Darmstadt, Germany) was used for column chromatography. The packing material for molecular sieve column chromatography was Sephadex LH-20 (Pharmacia, Uppsala, Sweden). Precoated silica gel F_254_ plates and RP-C_18_ F_254s_ plates (Merck) were used for thin-layer chromatography (TLC). Spots were detected after TLC under UV light or by heating after spraying with anisaldehyde sulfuric acid.

### 3.2. Plant Material

The leaves of *Ginkgo biloba* were collected at the arboretum of Sungkyunkwan University, Suwon, Korea, in August 2016. The plant material was authenticated by one of the authors (K. H. Kim). A voucher specimen of the material (UHY-2016) was deposited in the herbarium of the School of Pharmacy, Sungkyunkwan University, Suwon, Republic of Korea.

### 3.3. Extraction and Isolation

Air drying of *G. biloba* leaves was performed in a room at approximately 25 °C with humidity levels between 20 and 30%. The drying process lasted for a week and was conducted without direct sunlight exposure. Dried leaves of *G. biloba* (1.7 kg) were extracted with 80% aqueous MeOH (3.0 L × 3) and filtered. The extract was concentrated under vacuum using a rotary evaporator to gain a crude MeOH extract (293.8 g). The extract was suspended in distilled water (700 mL) and solvent-partitioned with hexane, dichloromethane (CH_2_Cl_2_), ethyl acetate (EtOAc), and *n*-butanol (*n*-BuOH), and then, four fractions were gained: hexane-soluble (31.2 g), CH_2_Cl_2_-soluble (5.5 g), EtOAc-soluble (7 g), and *n*-BuOH-soluble fractions (37.4 g). The hexane-soluble fraction (31.2 g) was subjected to silica gel column chromatography, employing a gradient solvent system of CH_2_Cl_2_/MeOH (100:1 to 1:1). Subsequently, the column was eluted with MeOH to yield six fractions (H_A_–H_F_). Fraction H_D_ (5.6 g) was fractionated by MPLC on the Universal ODS-SM column with a gradient solvent system of MeOH/H_2_O (65% MeOH to 75% MeOH) to afford four subfractions (H_D_1–H_D_4). Subfraction H_D_1 (171 mg) was subjected to silica gel column chromatography, employing a gradient solvent system of CH_2_Cl_2_/MeOH (20:1 to 1:1) to obtain eight subfractions (H_D_11–H_D_18). Subfraction H_D_13 (9.9 mg) was purified by semi-preparative HPLC with an isocratic solvent system of MeCN/H_2_O (27% MeCN; acetonitrile) to yield compound **6** (*t*_R_ = 61.2 min, 0.5 mg). Subfraction H_D_2 (87.6 mg) was separated using preparative reversed-phase HPLC with a gradient solvent system of MeOH/H_2_O (67% MeOH to 100% MeOH) to obtain four subfractions (H_D_21–H_D_24). Subfraction H_D_24 (40.5 mg) was purified by semi-preparative HPLC with an isocratic solvent system of MeCN/H_2_O (48% MeCN) to yield compounds **2** (*t*_R_ = 39.5 min, 0.9 mg) and **3** (*t*_R_ = 42.5 min, 0.8 mg). Fraction H_E_ (4.3 g) was fractionated by Sephadex LH-20 column chromatography using a solvent system of 100% MeOH to yield four subfractions (H_E_1–H_E_4). Subfraction H_E_3 (110.1 mg) was separated using preparative reversed-phase HPLC with a gradient solvent system of MeOH/H_2_O (40% MeOH to 90% MeOH) to afford five subfractions (H_E_31–H_E_35). Subfraction H_E_33 (7.0 mg) was purified using semi-preparative HPLC with an isocratic solvent system of MeCN/H_2_O (27% MeCN) to isolate compound **4** (*t*_R_ = 21.2 min, 0.7 mg). Subfraction H_E_34 (13.1 mg) was purified using semi-preparative HPLC with an isocratic solvent system of MeCN/H_2_O (45% MeCN) to obtain compound **5** (*t*_R_ = 23.8 min, 1.3 mg). Subfraction H_E_35 (30.7 mg) was purified using semi-preparative HPLC with an isocratic solvent system of MeCN/H_2_O (48% MeCN) to obtain compound **1** (*t*_R_ = 25.6 min, 1.2 mg). The scheme of isolation process is described in Figure S19.

#### (*aS*)-Glucosciadopitysin (**1**)

Yellowish plate; ${\left[\alpha \right]}_{D}^{20}$ −42.8 (*c* 0.01, MeOH); UV (MeOH) *λ*_max_ (log ε) 205 (3.9), 270 (2.9), 330 (2.8) nm (Figure S2); ECD (0.6 mg/mL, MeOH) *λ*_max_ (∆ε) 221 (−1.07), 241 (0.22), 267 (−1.30), 306 (−1.49), 335 (2.95) nm; IR (neat) v_max_: 3436 (alcoholic O–H), 1664 (conjugated C=O), 1607 (C=C), 1506 (C=C), 1445 (C-C), 1259 (C_ar_-O) cm^−1^; ^1^H (850 MHz) and ^13^C (212.5 MHz) NMR data, see Table 1; HR-ESIMS (positive ion mode) *m/z* 743.1977 [M+H]^+^ (calcd. for C_39_H_35_O_15_^+^, 743.1970).

### 3.4. Acid Hydrolysis and Absolute Configuration Determination of the Sugar Moiety of **1**

An HPLC-UV-based method was used to confirm the absolute configuration of the sugar moiety of **1** [24]. Compound **1** (0.5 mg) was hydrolyzed by 1 N HCl (1 mL) at 80 °C for 1 h, and solvent partitioned with CH_2_Cl_2_ (1 mL) to separate the aglycone and sugar. The aqueous layer was neutralized by repeated evaporation using a vacuum evaporator and dissolved in anhydrous pyridine (0.5 mL) with the addition of L-cysteine methyl ester hydrochloride (0.5 mg). After the reaction mixture was heated at 60 °C for 1 h, *o*-tolyl isothiocyanate (30 μL) was added, and the mixture was kept at 60 °C for 1 h. The reaction product, amounting to 30 μL, was evaporated using a vacuum evaporator and dissolved in 300 μL MeOH. Then, 10 μL of the sample was injected and analyzed by LC/MS with a gradient solvent system [MeOH/H_2_O, 0% MeOH → 80% MeOH gradient system (0–31 min), 100% MeOH (31–41 min), and 0% MeOH (42–52 min)]. The sugar moiety from **1** was identified as *β*-D-glucose based on a comparison of the retention time with the standard *β*-D-glucose subjected to the derivatization (*t*_R_: 19.5 min).

### 3.5. Electronic Circular Dichroism (ECD) Calculations

Initial conformational searches were performed for compound **1** in the MMFF94 force field using the MacroModel (version 2021-4, Schrödinger LLC, New York, NY, USA) programme with a mixed torsional/low-mode sampling method, in which a gas phase with a 20 kJ/mol energy window and 10,000 maximum iterations were employed. The Polak–Ribiere conjugate gradient (PRCG) algorithm was established with 10,000 maximum iterations and a 0.001 kJ (mol Å)^−1^ convergence threshold on the root-mean-squared gradient to minimize conformers. The proposed conformers (found within 5 kJ/mol in the MMFF force field) were distinguished by their axial chirality and selected for geometry optimization using TmoleX 4.3.2, with density functional theory settings of B3-LYP/6-31+G (d,p) [33].

ECD calculations were conducted for each conformer of (*aS*)-**1** (10 conformers) and (*aR*)-**1** (10 conformers) at the theoretical level and basis sets. The calculated ECD spectra were simulated by superimposing each transition, where *σ* is the band width at a height of 1/e, and Δ*E_i_* and *R_i_* are the excitation energy and rotatory strength for transition *i*, respectively [33]. In this study, the value of *σ* was 0.2 eV. The excitation energies and rotational strengths of the ECD spectra were calculated based on the Boltzmann populations of conformers, and ECD visualization was performed using SigmaPlot 14.0.

$$∆\epsilon \left(E\right)=\frac{1}{2.297\times 10^{-39}}\frac{1}{\sqrt{2\pi \sigma }}\sum_{A}^{i}∆E_{i}R_{i}e^{\left[-{\left(E-∆E_{i}\right)}^{2}/{\left(2\sigma \right)}^{2}\right]}$$

### 3.6. Cell Culture and Differentiation

The C3H10T1/2 cell line was obtained from the American Type Culture Collection (ATCC, Manassas, VA, USA). The cells were maintained in Dulbecco’s Modified Eagle’s Medium (DMEM, Hyclone Laboratories Inc., Logan, UT, USA) supplemented with 10% fetal bovine serum (FBS) (Hyclone) and antibiotics (100 U/mL of penicillin and 100 μg/mL of streptomycin, Hyclone) at 37 °C in a 5% CO_2_ humidified atmosphere. For adipocyte differentiation, confluent cells were cultured in DMEM supplemented with 10% FBS, 1 μM dexamethasone (Sigma, St. Louis, MO, USA), 0.5 mM isobutyl-1-methylxanthine (Sigma), 5 μg/mL insulin (Sigma), and 10 μM troglitazone for 48 h. Subsequently, the cells were transferred to DMEM containing 10% FBS, 5 μg/mL insulin, and 10 μM troglitazone and further incubated for an additional 3 days. During adipogenesis, 20 µM of compounds **1–6** was added to the cells, and resveratrol (20 µM) was used as a positive control. Resveratrol (CAS No. 501-36-0) was purchased from Sigma-Aldrich (Cat. No. R5010). For osteogenic differentiation, cells reaching confluence were cultured in DMEM supplemented with 10% FBS, 100 U/mL of penicillin, and 100 μg/mL of streptomycin, along with 10 mM β-glycerophosphate (Sigma-Aldrich) and 50 μg/mL ascorbic acid (Sigma-Aldrich), for 9 to 12 days. The medium was refreshed every 3 days. During osteogenic differentiation, 20 µM of compounds **1–6** was added to the cells, and 5 µM oryzativol A was used as a positive control.

### 3.7. Oil Red O Staining

The cultured cells were rinsed with phosphate-buffered saline and fixed in 10% neutral-buffered formalin at room temperature for 1 h. Subsequently, the cells were stained with a 0.5% filtered stock solution of Oil Red O in isopropanol (Sigma, Saint Louis, MO, USA). After staining, the intracellular triglyceride content was assessed by redissolving the stained cells in isopropanol, followed by a measurement of absorbance at a wavelength of 520 nm.

### 3.8. Alkaline Phosphatase (ALP) Staining

The cultured cells were rinsed with 2 mM MgCl_2_ solution. Subsequently, they were incubated with ALP buffer (composed of 100 mM Tris-HCl, pH 9.5; 100 mM NaCl; 10 mM MgCl_2_; and 0.05% Tween-20) for 15 min. Following this, the cells were incubated in ALP buffer supplemented with 0.4 mg/mL of nitro-blue tetrazolium (Merck) and 0.2 g/mL of 5-bromo-4-chloro-3-indolyl phosphate (Merck). After washing with 0.5 mM ethylenediaminetetraacetic acid, the cells were fixed with 10% neutral-buffered formalin for 1 h.

### 3.9. ALP Activity

To assess ALP activity, an alkaline phosphatase assay kit (ab83369; Abcam, Cambridge, MA, USA) was employed. Cell lysates obtained from the differentiated osteogenic cells were treated with a p-nitrophenyl phosphate (*p*-NPP) solution at 25 °C for 1 h in the dark. Following cessation of the reaction, the absorbance of the samples was measured at 405 nm.

### 3.10. mRNA Isolation and Real-Time Polymerase Chain Reaction (PCR)

RNA extraction from the cells was performed using the NucleoZOL reagent (NucleoZOL; Macherey-Nagel GmbH & Co., KG, Düren, Germany). Subsequently, complementary DNA (cDNA) was synthesized from 0.5 μg of total RNA utilizing a ReverTraAce qPCR reverse transcription (RT) Master Mix Kit (FSQ-201; Toyobo, Japan) with random primers. The resulting cDNA was then combined with the amplification mixture comprising the Thunderbird SYBR qPCR Mix (Toyobo) along with the specified primers.

Alkaline phosphatase (*ALP*): forward 5′-CAAGGATGCTGGGAAGTCCG-3′ and reverse 5′-CGGATAACGAGATGCCACCA-3′; osteopontin (*OPN*): forward 5′-CTGGCAGCTCAGAGGAGAAG-3′ and reverse 5′-CAGCATTCTGTGGCGCAAG-3′.

### 3.11. Statistical Analysis

Each sample was tested in triplicate, and the test was repeated three times. Data are presented as the mean ± standard deviation. One-way analysis of variance was used to identify statistically significant differences between the control and test groups.

## 4. Conclusions

The phytochemical investigation of the MeOH extract from *G. biloba* leaves led to the isolation and identification of six flavonoids, including a new biflavonoid glycoside, (*aS*)-glucosciadopitysin (**1**). The chemical structure of (*aS*)-glucosciadopitysin (**1**), along with its absolute configuration, was fully confirmed through comprehensive analyses involving 1D and 2D NMR, HR-ESIMS, acid hydrolysis, and computational methods for ECD calculation. Our study also evaluated the effects of isolated compounds on MSC differentiation into adipocytes and osteoblasts, revealing that ginkgetin (**2**) markedly enhanced osteoblastic differentiation, as demonstrated by its ability to stimulate ALP production in a concentration-dependent manner and promote the mRNA expression of osteogenic markers such as *ALP* and *OPN*. These findings are particularly significant, suggesting the potential therapeutic efficacy of ginkgetin (**2**) in preventing and treating conditions such as osteoporosis and other bone loss disorders. In future research, we will explore the osteogenic efficacy of ginkgetin (**2**) within an in vivo animal model of osteoporosis. Additionally, we will evaluate the safety profile of ginkgetin (**2**). The molecular mechanisms underlying its osteogenic activity will be investigated by examining key signalling pathways, such as Wnt, BMP, and Runx2, to assess ginkgetin’s therapeutic potential for treating osteoporosis.

## Figures and Tables

**Table 1:** ^1^H (850 MHz) and ^13^C NMR (212.5 MHz) data of compound **1** (CD_3_OD, *δ* in ppm) and ^1^H (400 MHz) and ^13^C NMR (100 MHz) literature data of sciadopitysin, an aglycone of compound **1** (Pyridine-*d*_5_, *δ* in ppm).

Position	1		Sciadopitysin [23]	
	*δ*_C_ ^a^, Type	*δ*_H_, (*J* in Hz)	*δ*_C_, Type	*δ*_H_, (*J* in Hz)
1				
2	164.5, C		164.3, C	
3	103.4, CH	6.79, s	104.1, CH	6.97, s
4	182.5, C		182.8, C	
5	161.5, C		161.5, C	
6	97.9, CH	6.35, d (2.0)	98.6, CH	6.58, d (2.4)
7	165.9, C		165.8, C	
8	91.9, CH	6.66, d (2.0)	92.8, CH	6.70, d (2.0)
9	157.9, C		155.5, C	
10	104.8, C		104.9, C	
1′	121.5, C		123.4, C	
2′	131.2, CH	8.08, d (2.0)	131.9, CH	8.51, d (2.0)
3′	122.9, C		123.1, C	
4′	160.1, C		162.6, C	
5′	111.1, CH	7.35, d (8.5)	111.8, CH	7.36, d (8.8)
6′	128.1, CH	8.16, dd (8.5, 2.0)	128.4, CH	8.16, dd (8.8, 2.4)
1′′				
2′′	164.4, C		164.3, C	
3′′	102.6, CH	6.75, s	104.7, CH	7.11, s
4′′	181.0, C		182.9, C	
5′′	161.1, C		162.4, C	
6′′	98.5, CH	6.82, s	99.7, CH	6.91, s
7′′	160.4, C		163.3, C	
8′′	106.2, C		104.7, C	
9′′	ND ^b^		158.0, C	
10′′	105.3, C		105.8, C	
1′′′	122.9, C		123.8, C	
2′′′	127.6, CH	7.59, d (9.0)	128.1, CH	7.75, d (8.8)
3′′′	114.1, CH	6.92, d (9.0)	114.8, CH	7.00, d (8.8)
4′′′	163.2, C		162.8, C	
5′′′	114.1, CH	6.92, d (9.0)	114.8, CH	7.00, d (8.8)
6′′′	127.6, CH	7.59, d (9.0)	128.1, CH	7.75, d (8.8)
1⁗	100.1, CH	5.19, d (7.5)		
2⁗	73.1, CH	3.25, dd (9.0, 7.5)		
3⁗	76.6, CH	3.45, dd (9.5, 9.0)		
4⁗	69.7, CH	3.37, dd (9.5, 9.0)		
5⁗	76.9, CH	3.50, ddd (9.0, 5.5, 2.5)		
6⁗a	60.7, CH_2_	3.76, dd (12.0, 5.5)		
6⁗b		3.92, dd (12.0, 2.5)		
7-OCH_3_	55.0, CH_3_	3.87, s	56.0, CH_3_	3.90, s
4′-OCH_3_	54.9, CH_3_	3.81, s	55.8, CH_3_	3.75, s
4′′′-OCH_3_	54.5, CH_3_	3.80, s	55.3, CH_3_	3.63, s

^a 13^C NMR data were measured and assigned based on the HSQC and HMBC experiments. ^b^ ND: Not detected.

## Data Availability

Data are contained within the article and Supplementary Materials.

## Supplementary Materials

The following supporting information can be downloaded at https://www.mdpi.com/article/10.3390/plants14020261/s1, Figure S1: The HR-ESI-MS data (positive-ion mode) of **1**; Figure S2: The UV spectrum (A), HPLC-UV chromatographic data for isolation (B), and purity verification data (C) of **1**; Figure S3: The ^1^H NMR spectrum of **1** (CD_3_OD, 850 MHz); Figure S4: The ^1^H-^1^H COSY spectrum of **1**; Figure S5: The NOESY spectrum of **1**; Figure S6: The HSQC spectrum of **1**; Figure S7: The HMBC spectrum of **1**; Figure S8: The extracted ion chromatogram (positive-ion mode) of LC/MS for sugar analysis of **1**; Figure S9: The HPLC-UV chromatographic data for isolation (A), purity verification data (B), UV spectrum (C), and ESI-MS data (D) of **2**; Figure S10: The ^1^H NMR spectrum of **2** (CD_3_OD, 850 MHz); Figure S11: The HPLC-UV chromatographic data for isolation (A), purity verification data (B), UV spectrum (C), and ESI-MS data (D) of **3**; Figure S12: The ^1^H NMR spectrum of **3** (CD_3_OD, 850 MHz); Figure S13: The HPLC-UV chromatographic data for isolation (A), purity verification data (B), UV spectrum (C), and ESI-MS data (D) of **4**; Figure S14: The ^1^H NMR spectrum of **4** (CD_3_OD, 850 MHz); Figure S15: The HPLC-UV chromatographic data for isolation (A), purity verification data (B), UV spectrum (C), and ESI-MS data (D) of **5**; Figure S16: The ^1^H NMR spectrum of **5** (CD_3_OD, 850 MHz); Figure S17: The HPLC-UV chromatographic data for isolation (A), purity verification data (B), UV spectrum (C), and ESI-MS data (D) of **6**; Figure S18: The ^1^H NMR spectrum of **6** (DMSO-*d*_6_, 850 MHz); Figure S19: Isolation scheme of compounds from the hexane-soluble fraction; Table S1: The ^1^H NMR (CD_3_OD, 850 MHz) data of compounds **2**–**5**; Table S2: The ^1^H NMR (DMSO-*d*_6_, 850 MHz) data of compound **6**.
