# Supplementary material for: (aS)-Glucosciadopitysin, a New Biflavonoid Glycoside from the Leaves of Ginkgo biloba and Osteogenic Activity of Bioflavonoids
Source: Plants (Basel). 2025 Jan 17;14(2):261. doi: 10.3390/plants14020261 (PMC11768450; doi:10.3390/plants14020261)
Supplement: Supplementary file 1 [file plants-14-00261-s001.zip › plants-3298532-supplementary.pdf]

Supplementary data

(aS)-Glucosciadopitysin, a New Biflavonoid Glycoside from the Leaves of Ginkgo biloba and Osteogenic Activity of Bioflavonoids

Se Yun Jeong 1,†, Kwang Ho Lee 1,†, Seon Hee Kim 2, Min Hye Yang 3,*, Gakyung Lee 4,* and Ki Hyun Kim 1,*

[1] School of Pharmacy, Sungkyunkwan University, Suwon 16419, Republic of Korea; dlawktkark@naver.com (S.Y.J.); sholaly@naver.com (K.H.L.)

[2] Research Institute, Sungkyun Biotech Co., Ltd., Anyang 14118, Republic of Korea; seonhee31@gmail.com

[3] Department of Pharmacy, College of Pharmacy and Research Institute for Drug Development, Pusan National University, Busan 46241, Republic of Korea

[4] Department of Integrative Biological Sciences and Industry, Sejong University, Seoul 05006, Republic of Korea

* Correspondence: mhyang@pusan.ac.kr (M.H.Y.); lgg1025@sejong.ac.kr (G.L.); khkim83@skku.edu (K.H.K.); Tel.: +82-31-290-7700 (K.H.K.)

[†] These authors contributed equally to this study.

Supplementary data Contents:

Figure S1. The HR-ESI-MS data (positive-ion mode) of **1**……S3
Figure S2. The UV spectrum (A), HPLC-UV chromatographic data for isolation (B), and purity verification data (C) of **1**……S4
Figure S3. The ^{1}H NMR spectrum of **1** (CD_3OD, 850 MHz) ……S6
Figure S4. The ^{1}H-^{1}H COSY spectrum of **1**……S7
Figure S5. The NOESY spectrum of **1**……S8
Figure S6. The HSQC spectrum of **1**……S9
Figure S7. The HMBC spectrum of **1**……S10
Figure S8. The extracted ion chromatogram (positive-ion mode) of LC/MS for sugar analysis of **1**……S11
Figure S9. The HPLC-UV chromatographic data for isolation (A), purity verification data (B), UV spectrum (C), and ESI-MS data (D) of **2**……S12
Figure S10. The ^{1}H NMR spectrum of **2** (CD_3OD, 850 MHz) ……S14
Figure S11. The HPLC-UV chromatographic data for isolation (A), purity verification data (B), UV spectrum (C), and ESI-MS data (D) of **3**……S15
Figure S12. The ^{1}H NMR spectrum of **3** (CD_3OD, 850 MHz) ……S17
Figure S13. The HPLC-UV chromatographic data for isolation (A), purity verification data (B), UV spectrum (C), and ESI-MS data (D) of **4**……S18
Figure S14. The ^{1}H NMR spectrum of **4** (CD_3OD, 850 MHz) ……S20
Figure S15. The HPLC-UV chromatographic data for isolation (A), purity verification data (B), UV spectrum (C), and ESI-MS data (D) of **5**……S21
Figure S16. The ^{1}H NMR spectrum of **5** (CD_3OD, 850 MHz) ……S23
Figure S17. The HPLC-UV chromatographic data for isolation (A), purity verification data (B), UV spectrum (C), and ESI-MS data (D) of **6**……S24
Figure S18. The ^{1}H NMR spectrum of **6** (DMSO-d_6, 850 MHz) ……S26
Figure S19. Isolation scheme of compounds from the hexane-soluble fraction ……S27

Table S1. The ^{1}H NMR (CD_3OD, 850 MHz) data of compounds **2–5** ……S28

Table S2. The ^{1}H NMR (DMSO-d_6, 850 MHz) data of compound **6** ……S33

Figure S1. The HR-ESI-MS data (positive-ion mode) of **1**

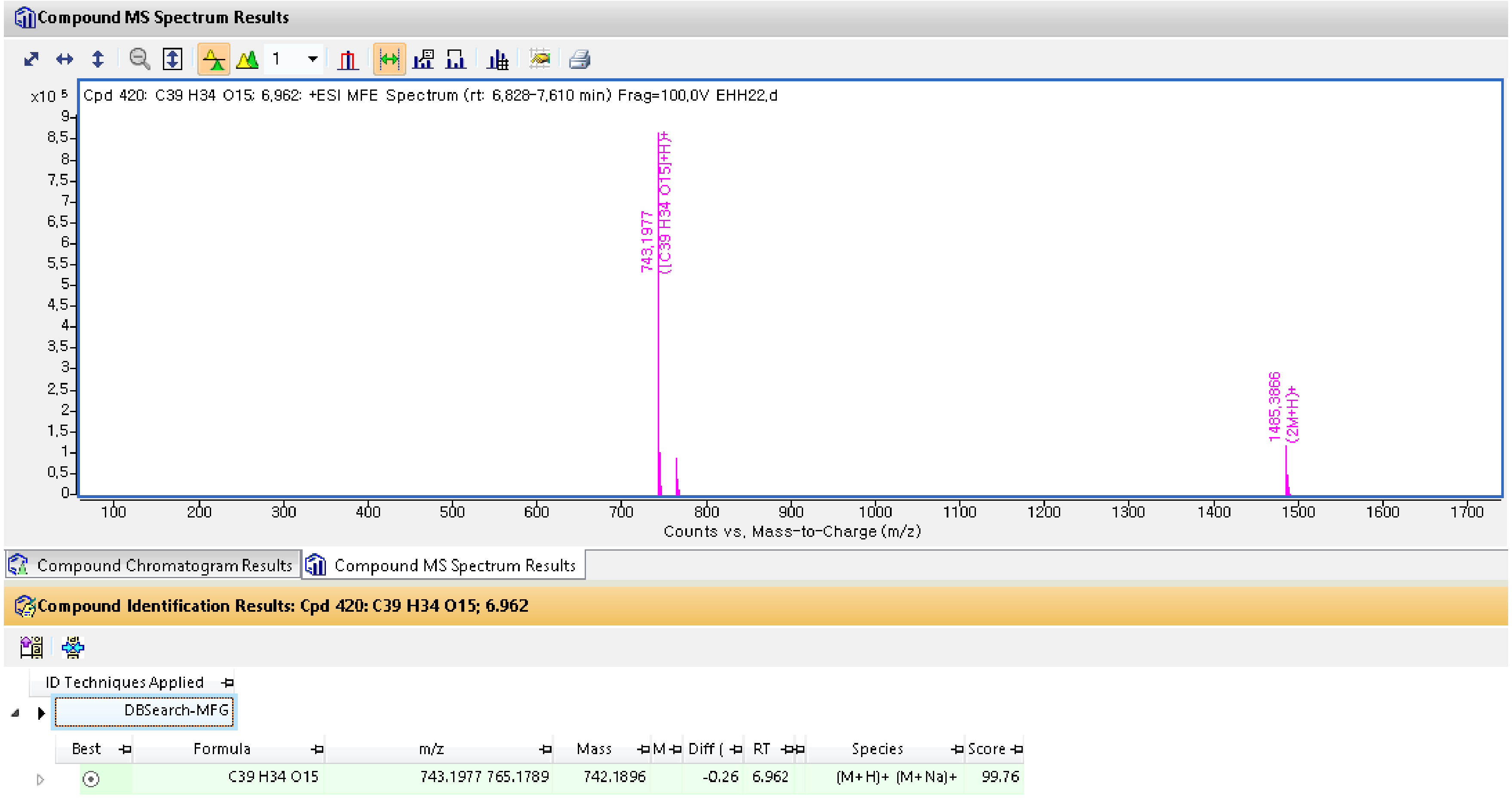

Figure S2. The UV spectrum (A), HPLC-UV chromatographic data for isolation (B), and purity verification data (C) of **1**

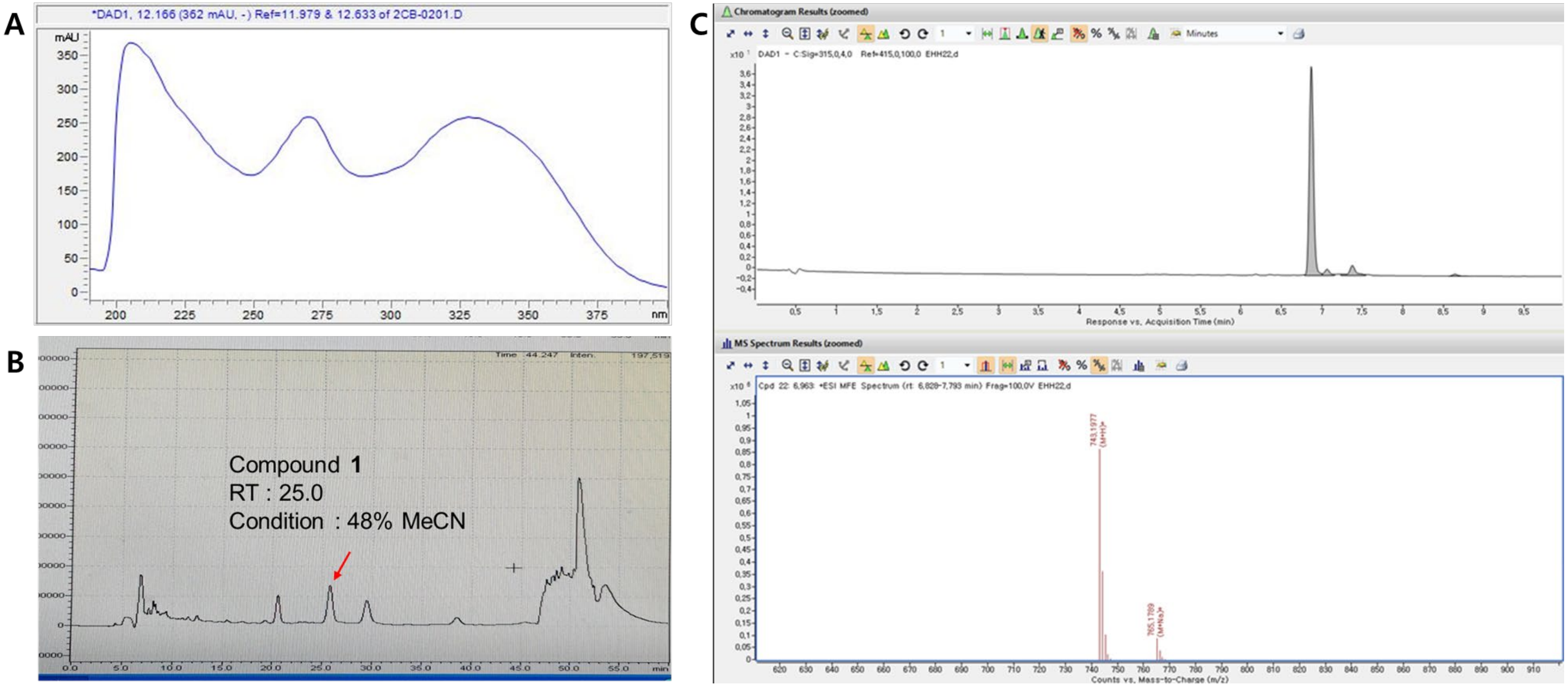

UV (MeOH) λ_{max} (log ε) 205 (3.9), 270 (2.9), 330 (2.8) nm;

1) 205 nm - This band typically represents the π → π* transitions in the benzene rings of the structure. At this short wavelength, the transition involves the excitation of electrons in the highly conjugated aromatic system. This peak is characteristic of transitions within the core benzene or phenyl chromophores.

2) 270 nm: This band is associated with π → π* transitions, specifically involving the conjugated aromatic rings and substituents such as hydroxyl or methoxy groups. The slightly lower intensity (log ε) compared to the 205 nm peak reflects the involvement of less strongly conjugated systems or weaker oscillator strength for this transition. This band can also indicate transitions in the flavonoid B-ring, which is often less conjugated than the rest of the system.

3) 330 nm: This band corresponds to n → π* transitions or extended conjugation involving the chromophores that connect the flavonoid units. It indicates electron excitation from non-bonding orbitals (typically oxygen in hydroxyl groups) to the π* orbitals of the aromatic system. This band is often shifted to higher wavelengths (bathochromic shift) due to conjugation between the two flavonoid units, highlighting the extensive π-electron delocalization.

Figure S3. The ^{1}H NMR spectrum of **1** (CD_3OD, 850 MHz)

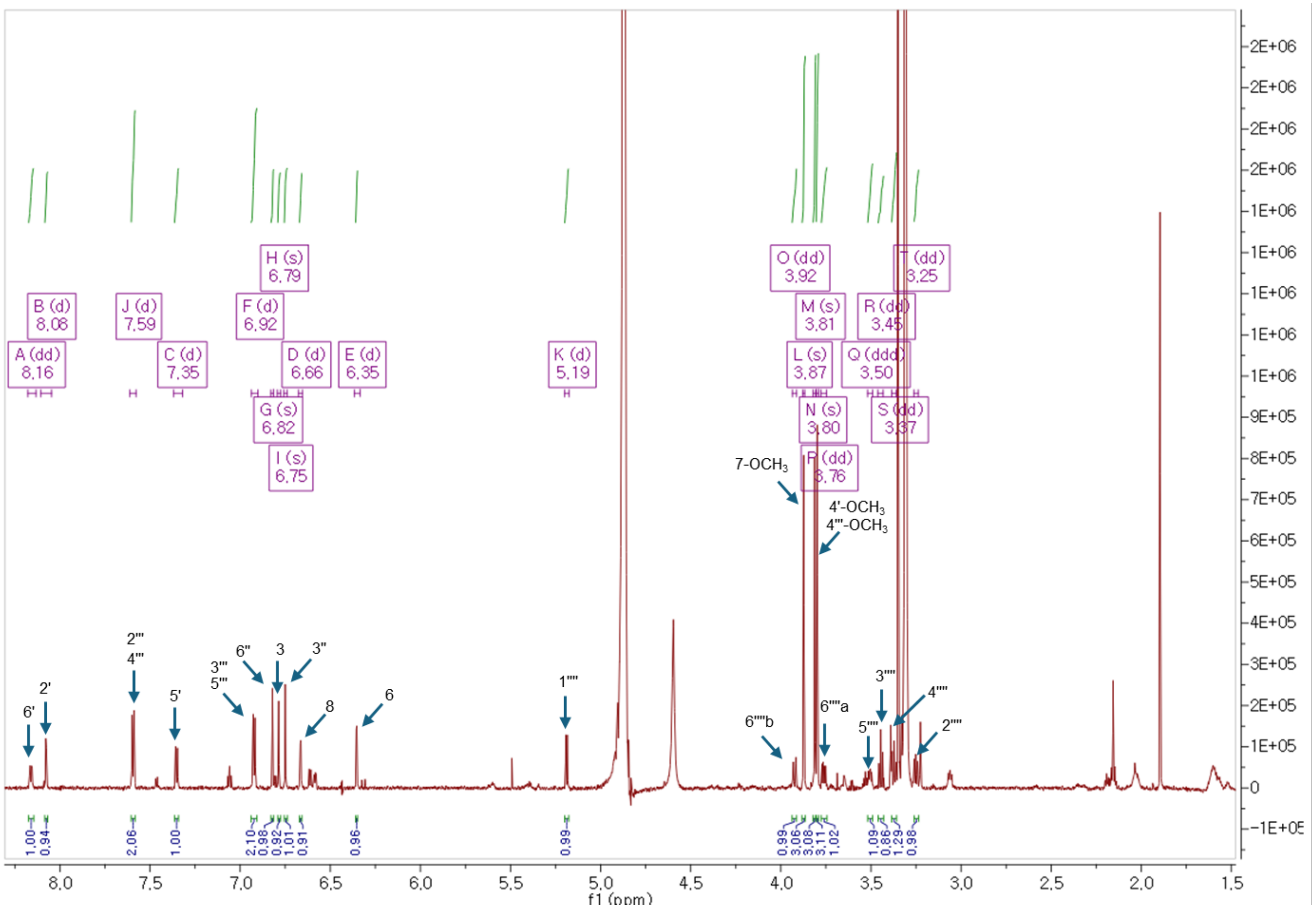

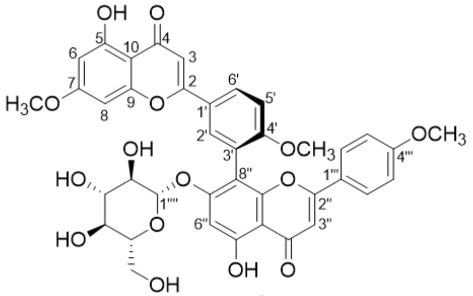

1

Figure S4. The ^{1}H-^{1}H COSY spectrum of **1**

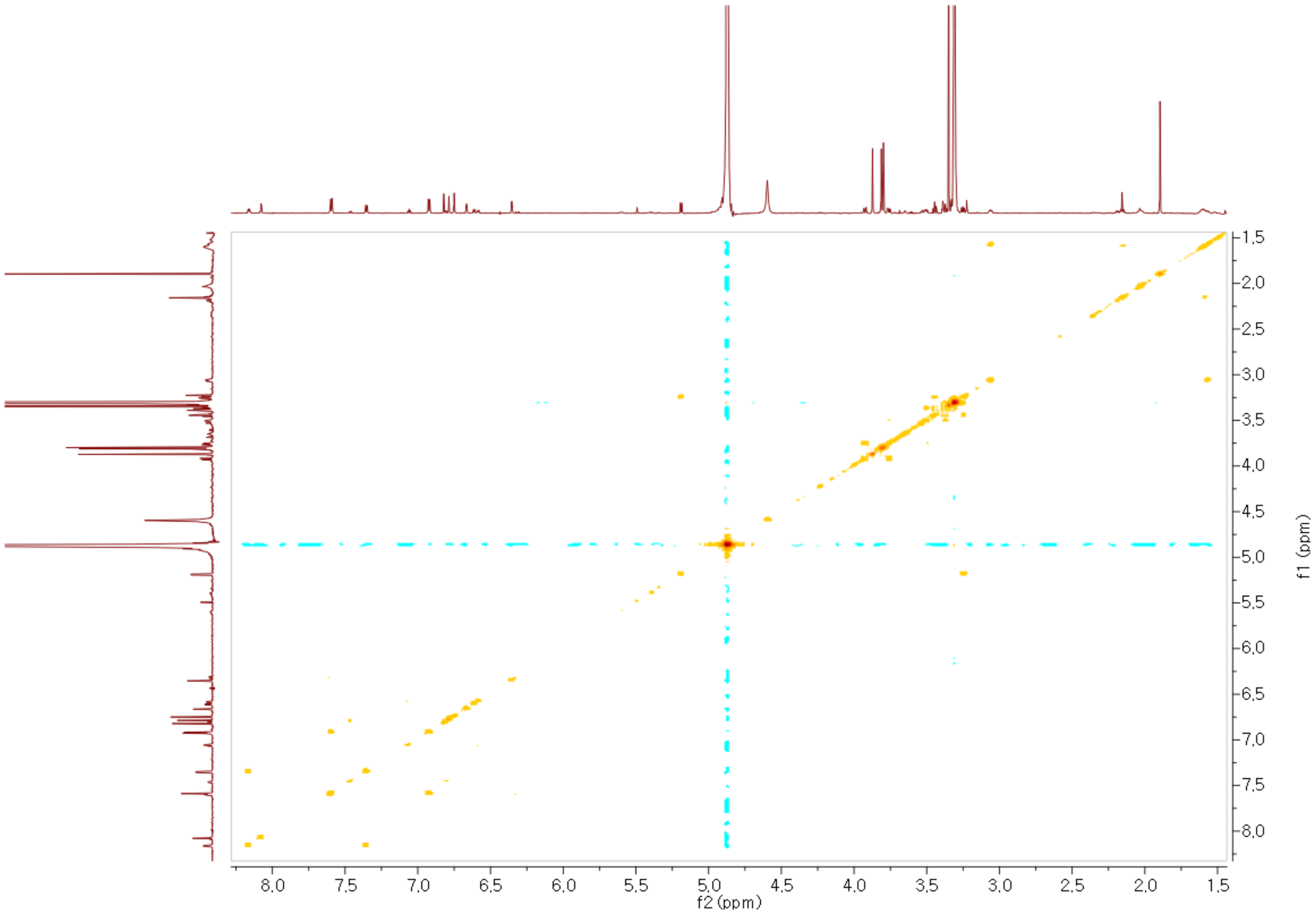

Figure S5. The NOESY spectrum of **1**

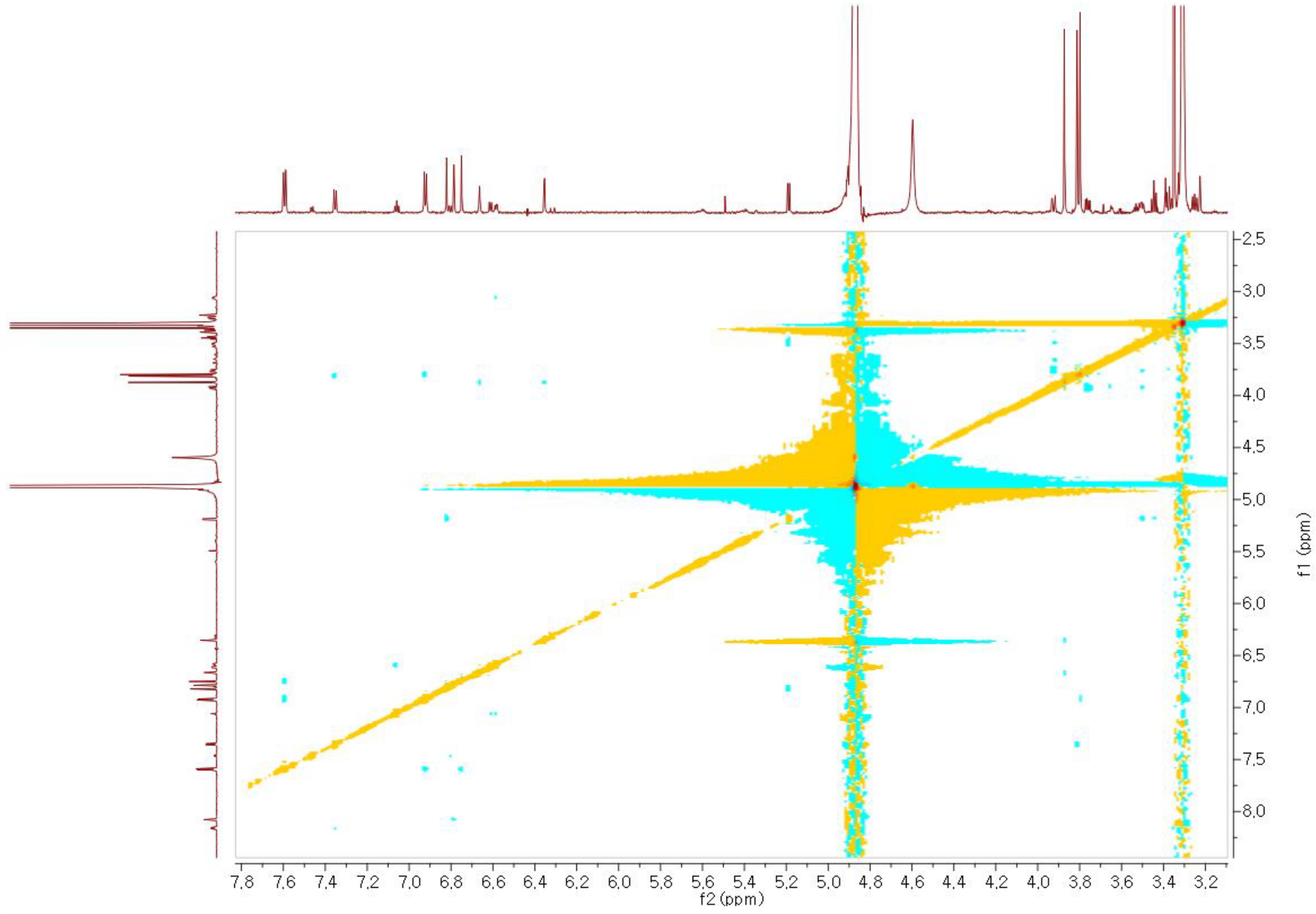

Figure S6. The HSQC spectrum of **1**

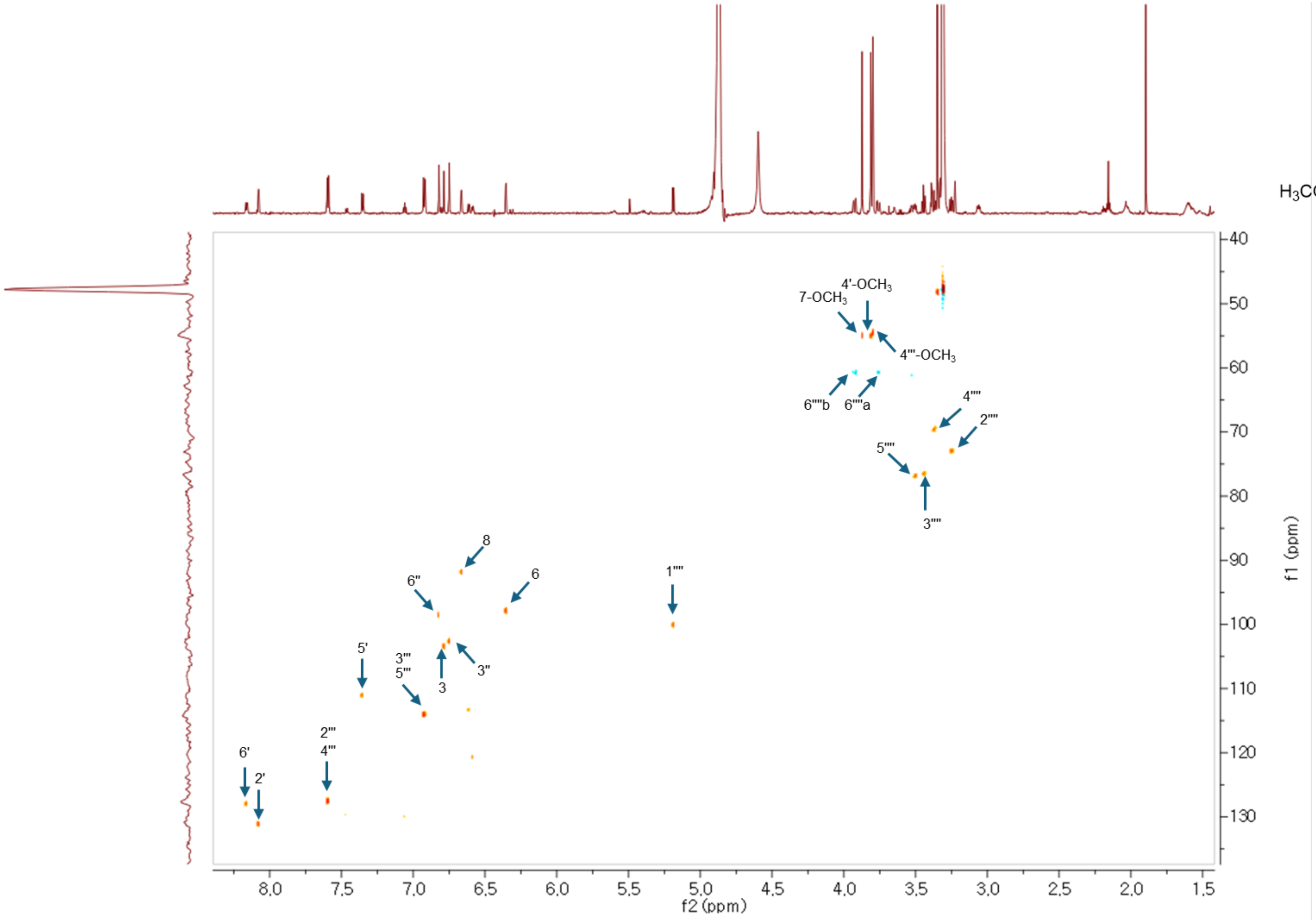

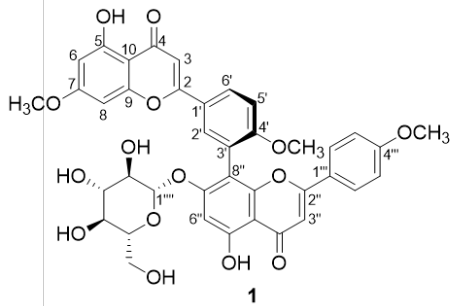

1

Figure S7. The HMBC spectrum of **1**

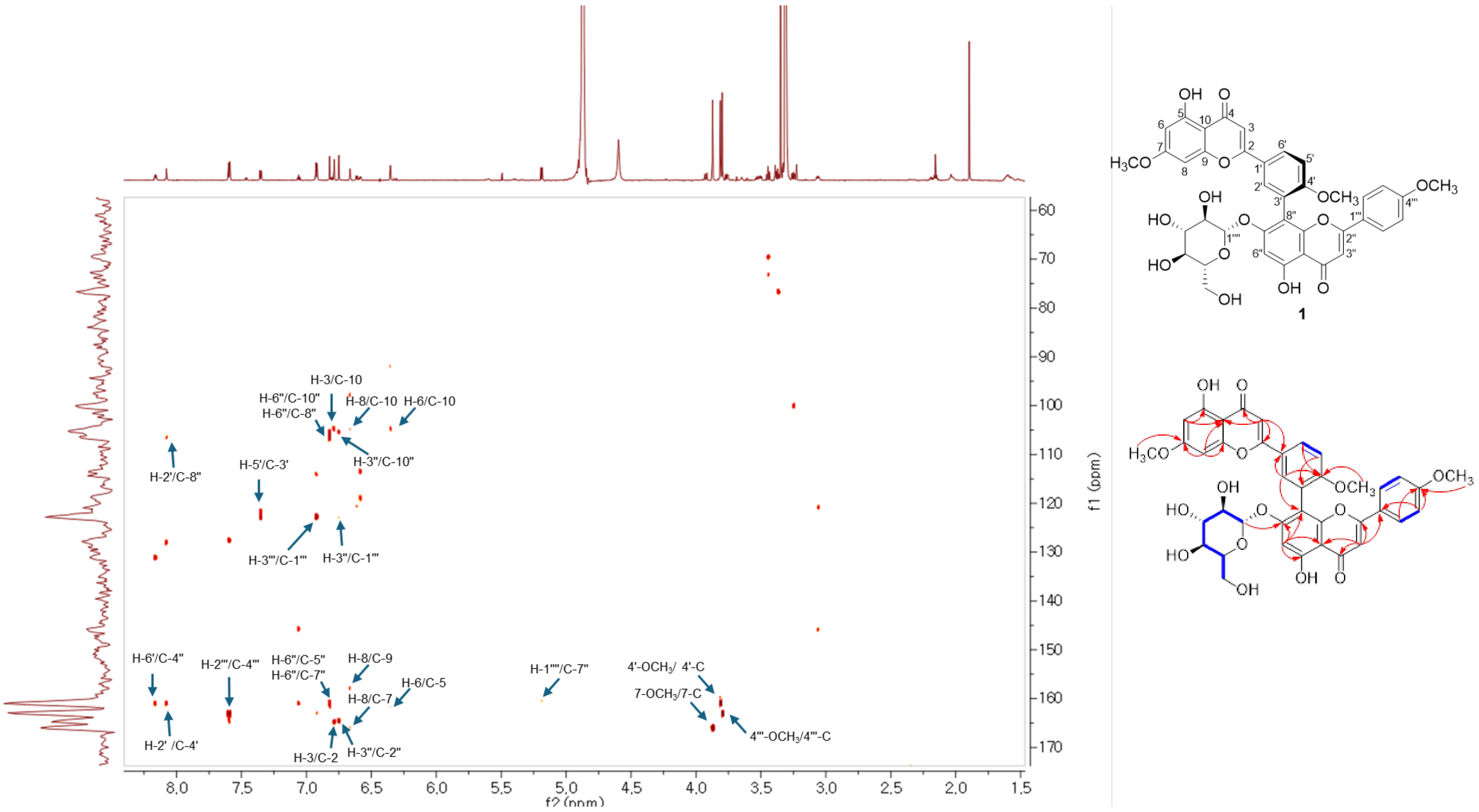

Figure S8. The extracted ion chromatogram (positive-ion mode) of LC/MS for sugar analysis of **1**

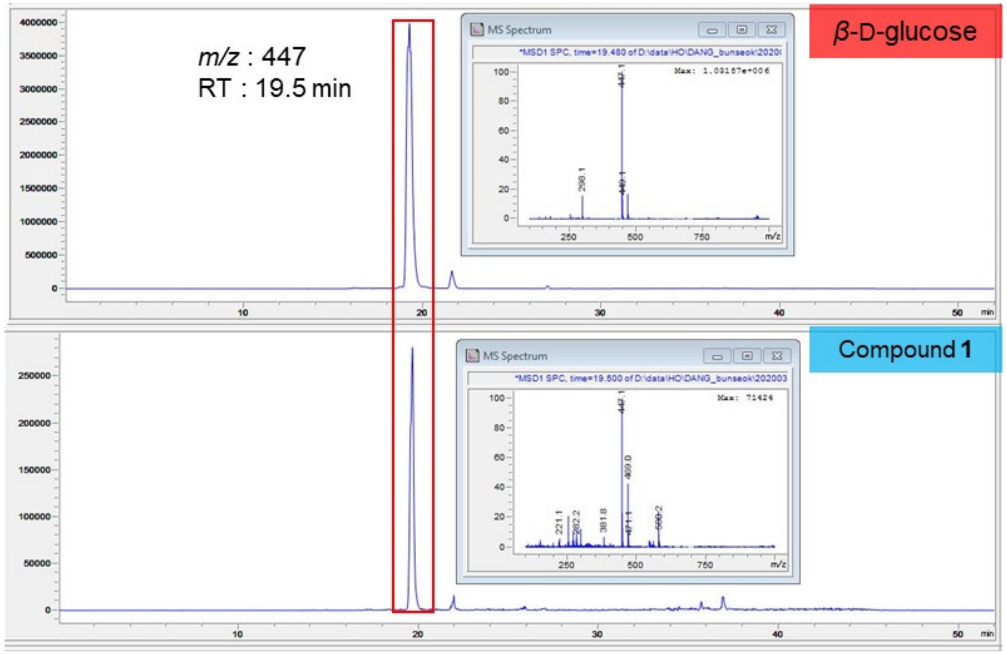

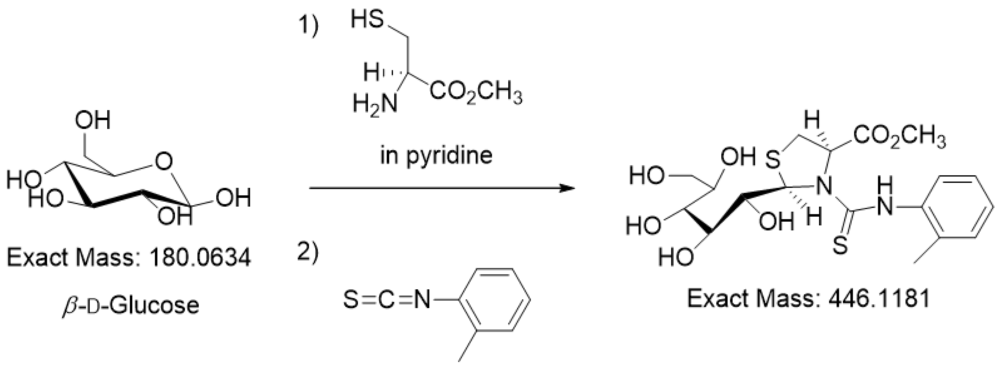

Figure S9. The HPLC-UV chromatographic data for isolation (A), purity verification data (B), UV spectrum (C), and ESI-MS data (D) of **2**

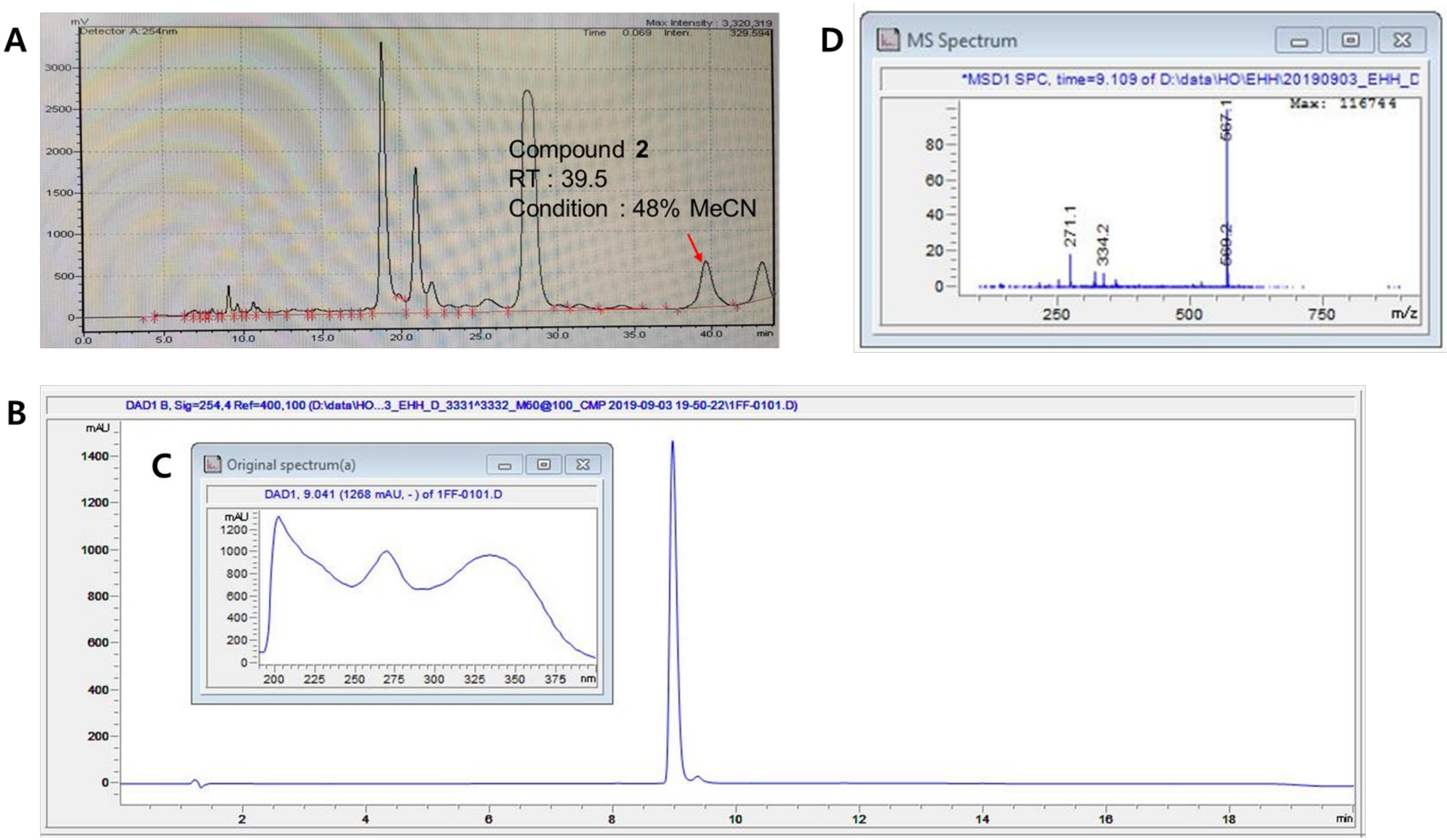

UV (MeOH) λ_{max} (log ε) 205 (3.9), 270 (2.9), 335 (2.8) nm;

1) 205 nm - This band typically represents the π → π* transitions in the benzene rings of the structure. At this short wavelength, the transition involves the excitation of electrons in the highly conjugated aromatic system. This peak is characteristic of transitions within the core benzene or phenyl chromophores.

2) 270 nm: This band is associated with π → π* transitions, specifically involving the conjugated aromatic rings and substituents such as hydroxyl or methoxy groups. The slightly lower intensity (log ε) compared to the 205 nm peak reflects the involvement of less strongly conjugated systems or weaker oscillator strength for this transition. This band can also indicate transitions in the flavonoid B-ring, which is often less conjugated than the rest of the system.

3) 335 nm: This band corresponds to n → π* transitions or extended conjugation involving the chromophores that connect the flavonoid units. It indicates electron excitation from non-bonding orbitals (typically oxygen in hydroxyl groups) to the π* orbitals of the aromatic system. This band is often shifted to higher wavelengths (bathochromic shift) due to conjugation between the two flavonoid units, highlighting the extensive π-electron delocalization.

Figure S10. The ^{1}H NMR spectrum of **2** (CD_3OD, 850 MHz)

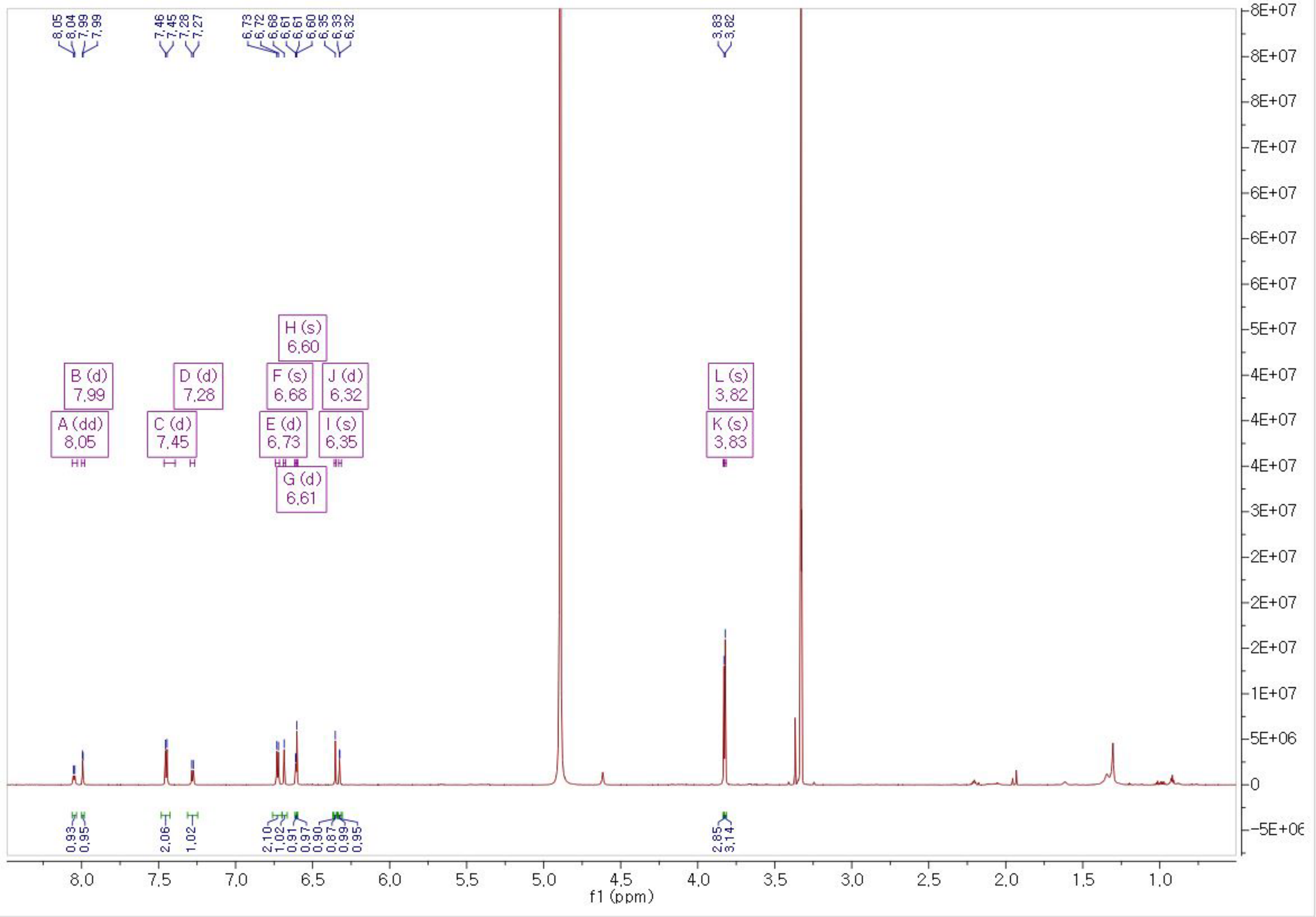

Figure S11. The HPLC-UV chromatographic data for isolation (A), purity verification data (B), UV spectrum (C), and ESI-MS data (D) of **3**

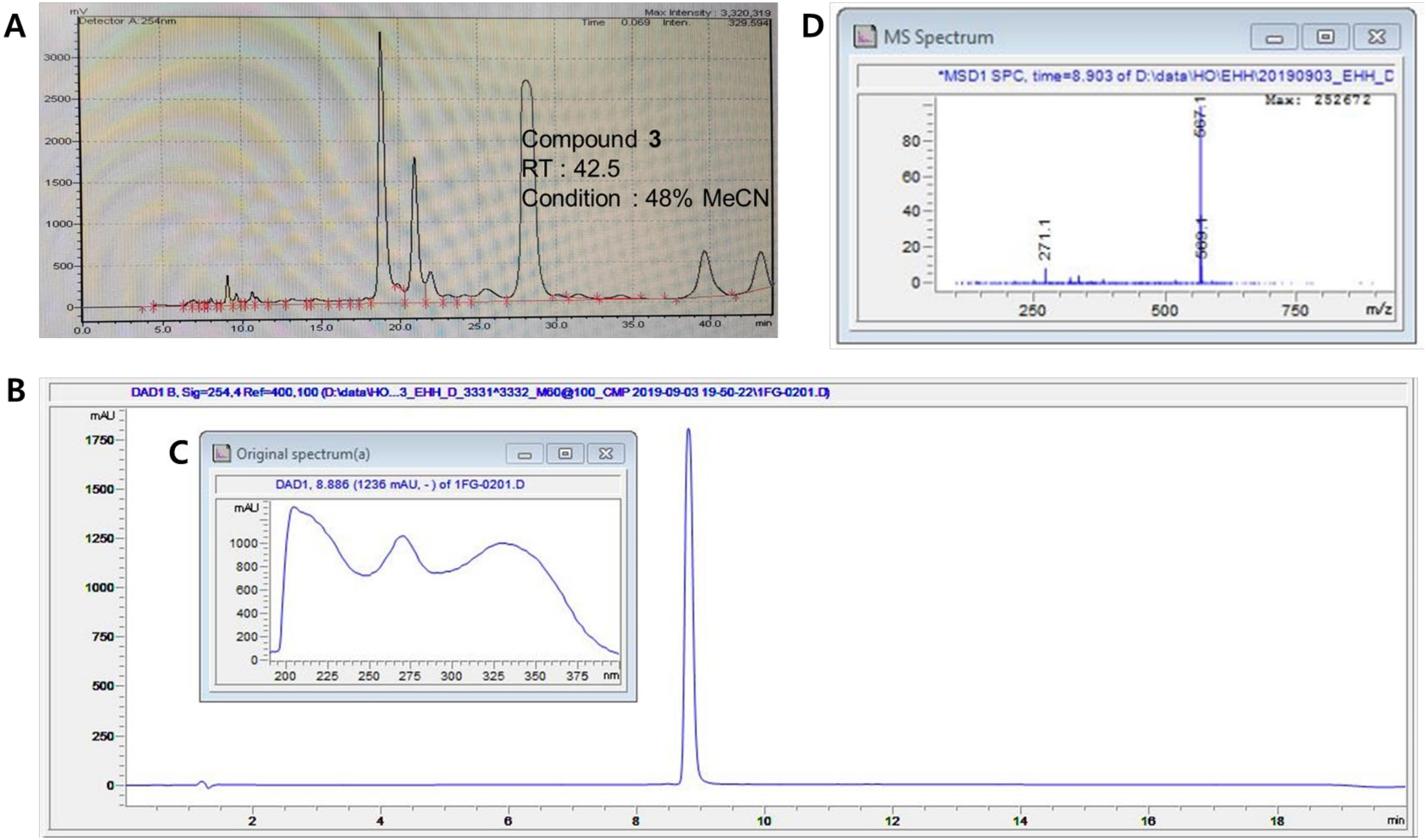

UV (MeOH) λ_{max} (log ε) 205 (3.9), 270 (2.9), 330 (2.8) nm;

1) 205 nm - This band typically represents the π → π* transitions in the benzene rings of the structure. At this short wavelength, the transition involves the excitation of electrons in the highly conjugated aromatic system. This peak is characteristic of transitions within the core benzene or phenyl chromophores.

2) 270 nm: This band is associated with π → π* transitions, specifically involving the conjugated aromatic rings and substituents such as hydroxyl or methoxy groups. The slightly lower intensity (log ε) compared to the 205 nm peak reflects the involvement of less strongly conjugated systems or weaker oscillator strength for this transition. This band can also indicate transitions in the flavonoid B-ring, which is often less conjugated than the rest of the system.

3) 330 nm: This band corresponds to n → π* transitions or extended conjugation involving the chromophores that connect the flavonoid units. It indicates electron excitation from non-bonding orbitals (typically oxygen in hydroxyl groups) to the π* orbitals of the aromatic system. This band is often shifted to higher wavelengths (bathochromic shift) due to conjugation between the two flavonoid units, highlighting the extensive π-electron delocalization.

Figure S12. The ^{1}H NMR spectrum of **3** (CD_3OD, 850 MHz)

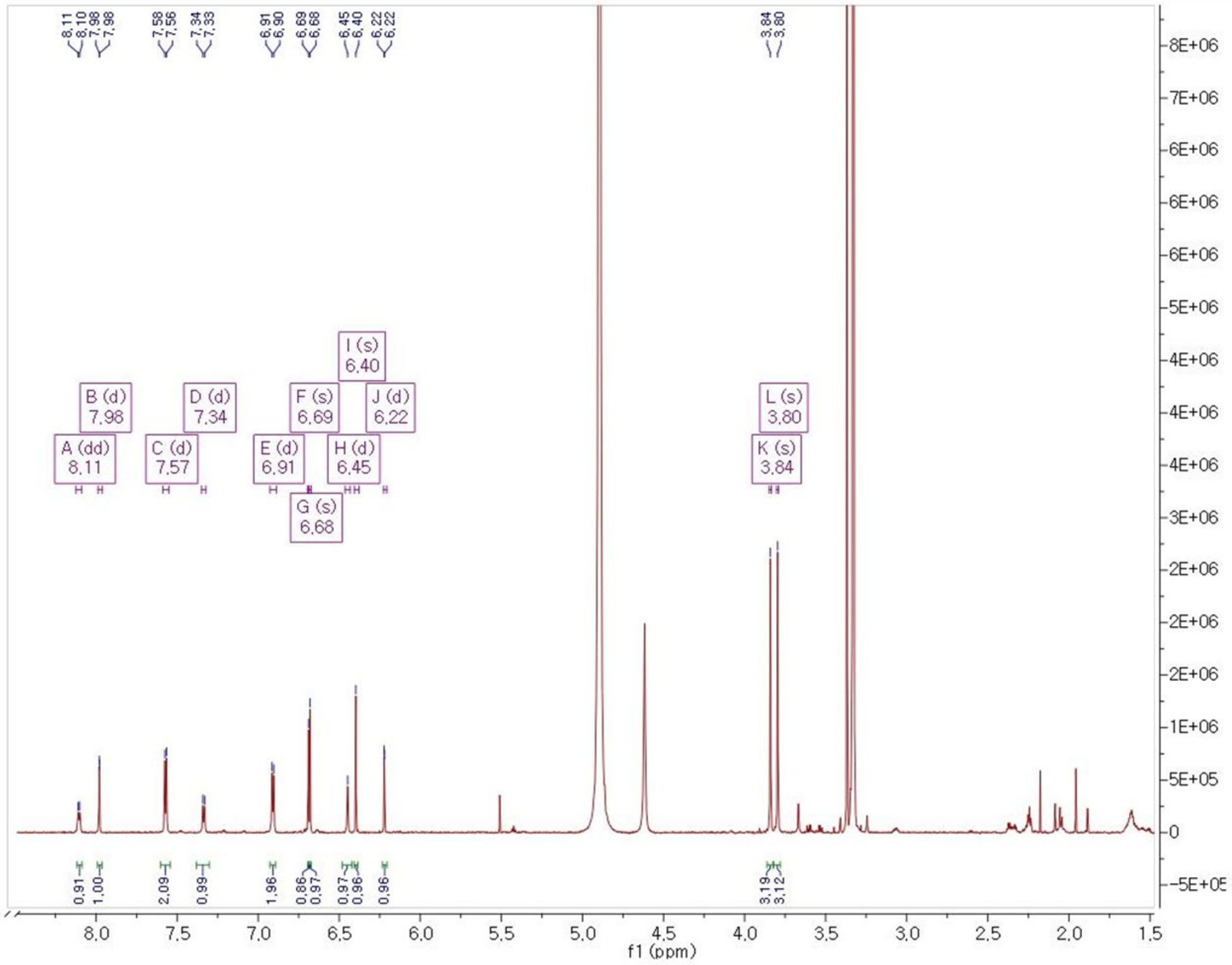

Figure S13. The HPLC-UV chromatographic data for isolation (A), purity verification data (B), UV spectrum (C), and ESI-MS data (D) of **4**

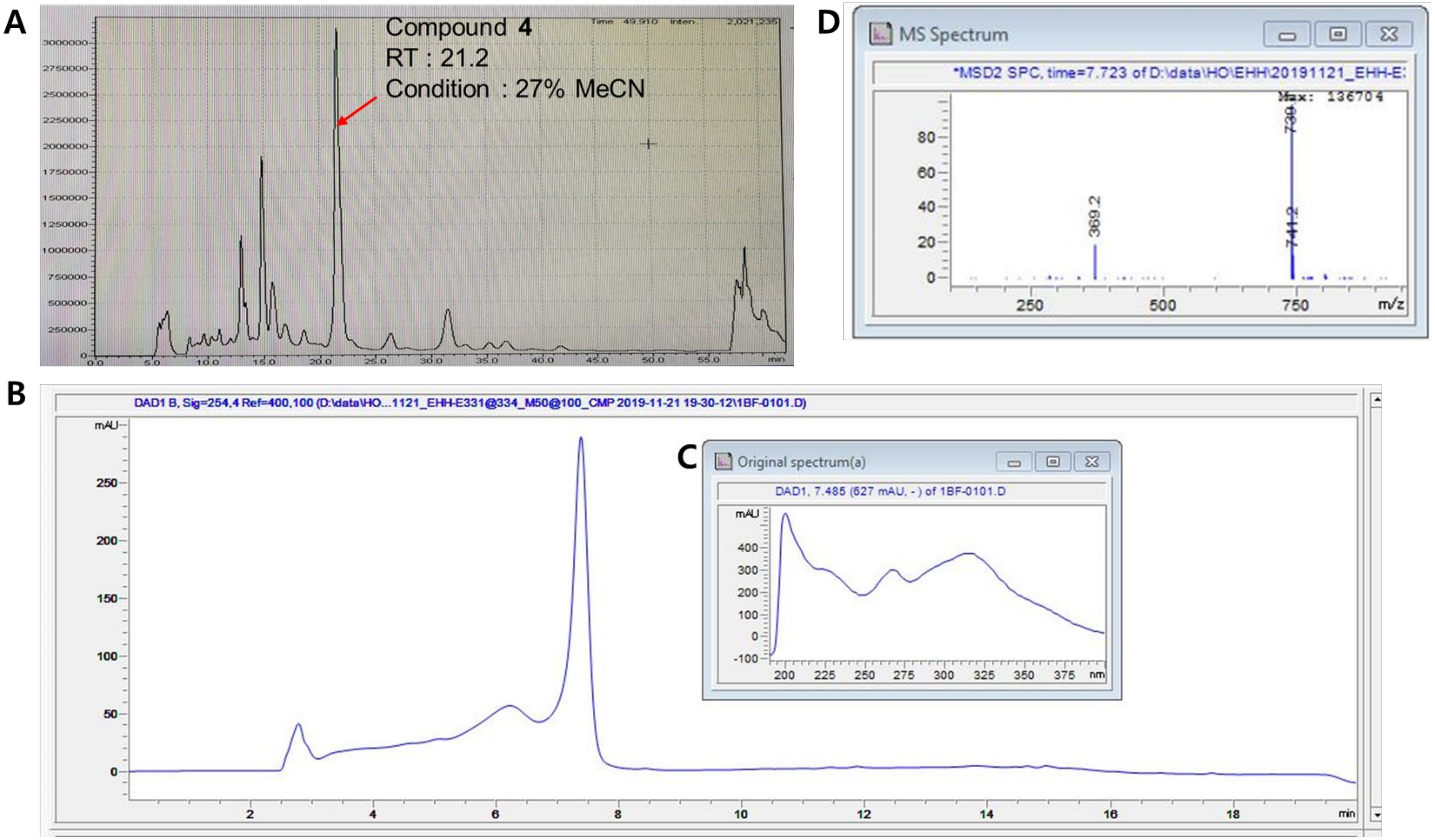

UV (MeOH) λ_{max} (log ε) 205 (4.0), 268 (2.7), 320 (3.3) nm;

1) 205 nm - This intense band is attributed to the π → π* transitions in the aromatic rings of the flavonoid backbone. This transition occurs within the benzene ring systems (A or B rings) and indicates localized excitation in these aromatic systems. The high intensity (log ε = 4.0) reflects a strong absorption due to the highly conjugated nature of these rings.

2) 268 nm: This band represents another π → π* transition, likely involving conjugation between the aromatic system and substituents such as hydroxyl or methoxy groups on the flavonoid structure. In glycosides, this peak may also reflect contributions from the glycosidic linkage or interaction of the sugar moiety with the chromophore. The lower intensity (log ε = 2.7) suggests a weaker transition compared to the band at 205 nm, possibly due to less extensive delocalization in this region.

3) 320 nm: This band corresponds to an n → π* transition or extended π → π* transitions involving conjugation between the C-ring (often containing a keto group) and the A and B rings of the flavonoid structure. In flavonoid glycosides, this band may exhibit a bathochromic shift due to interactions between the sugar and the chromophoric system, reflecting extended π-conjugation and hydrogen bonding. The moderate intensity (log ε = 3.3) indicates significant participation of conjugated systems in this transition.

Figure S14. The ^{1}H NMR spectrum of **4** (CD_3OD, 850 MHz)

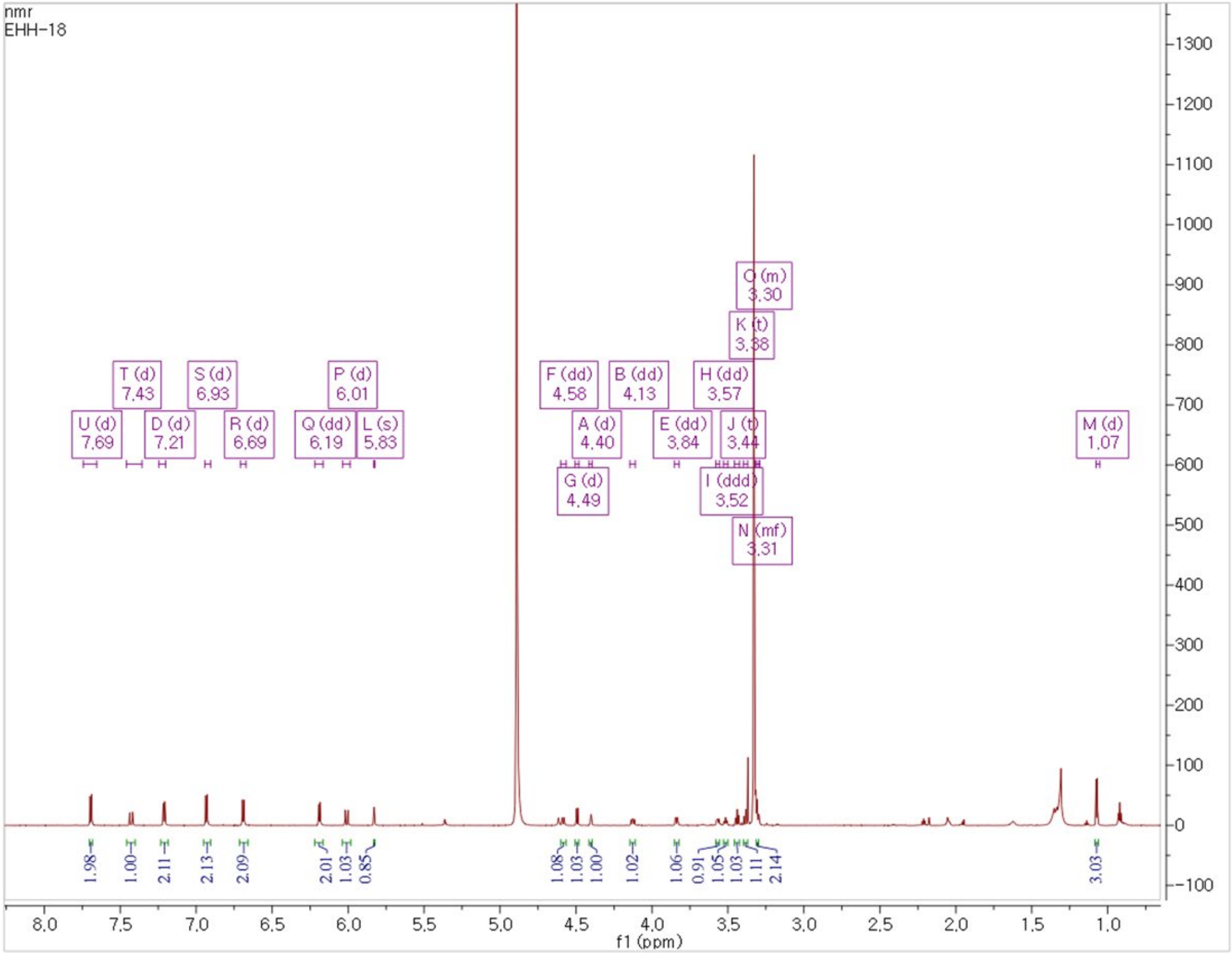

Figure S15. The HPLC-UV chromatographic data for isolation (A), purity verification data (B), UV spectrum (C), and ESI-MS data (D) of **5**

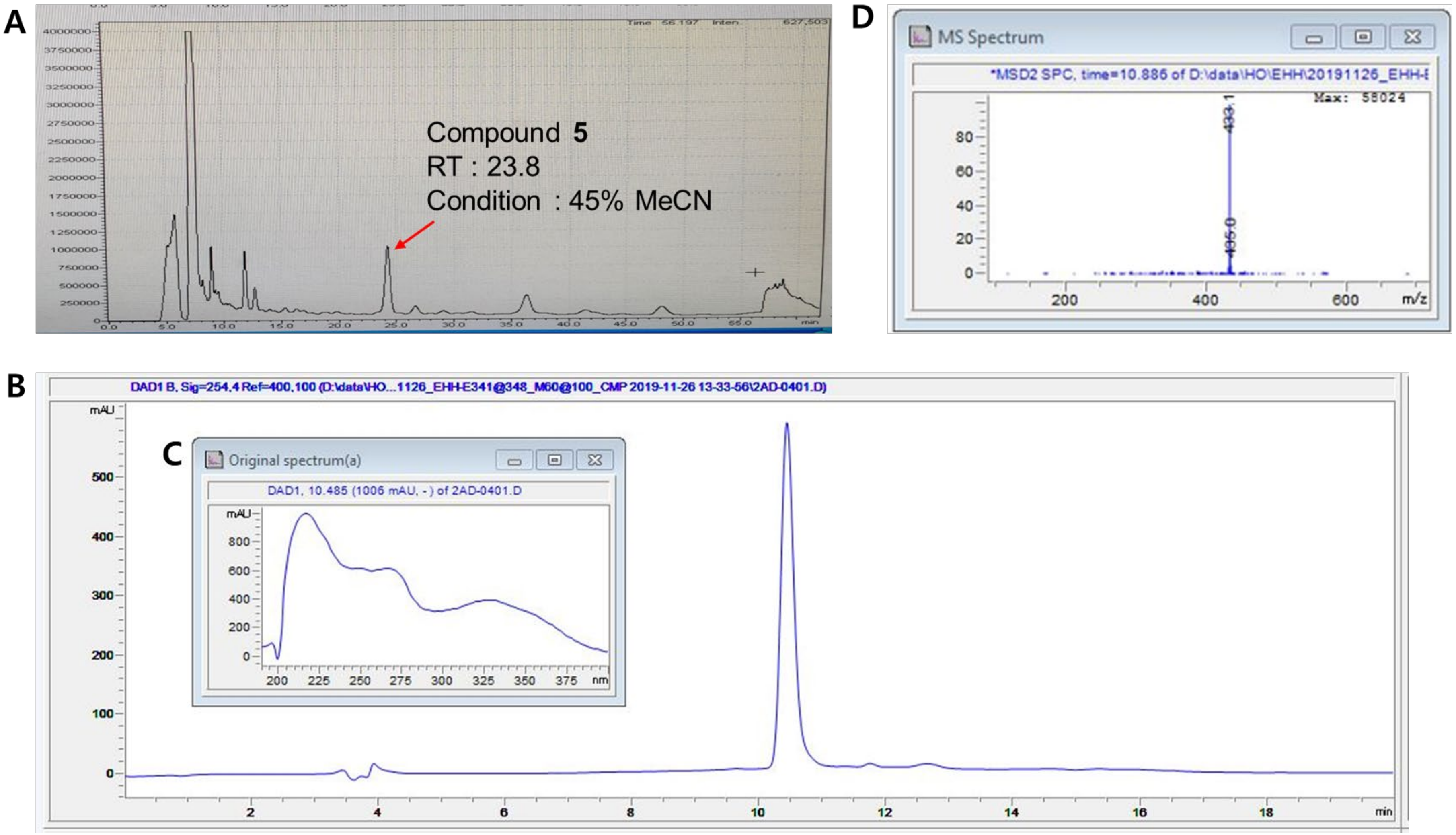

UV (MeOH) λ_{max} (log ε) 215 (4.2), 270 (2.7), 330 (2.3) nm;

1) 215 nm - This high-energy band is typically associated with the $\pi \rightarrow \pi^*$ transitions in the benzene ring. Flavonoids often have aromatic rings, and this absorption is attributed to the π-electrons in the conjugated system of the aromatic chromophores. The high molar absorptivity (log ε) indicates a strong transition.

2) 270 nm: This band represents another $\pi \rightarrow \pi^*$ transition, involving the conjugated C=C bond in the flavonoid backbone or aromatic system. It reflects conjugation between aromatic rings or unsaturated functional groups, and the lower intensity compared to 215 nm suggests a less allowed transition.

3) 330 nm: This band corresponds to an $n \rightarrow \pi^*$ transition, involving non-bonding electrons on oxygen atoms (e.g., carbonyl or hydroxyl groups) in the flavonoid structure. This absorption is indicative of charge transfer within conjugated chromophores, such as between the aromatic rings and carbonyl groups, often present in flavonoid molecules.

Figure S16. The ^{1}H NMR spectrum of **5** (CD_3OD, 850 MHz)

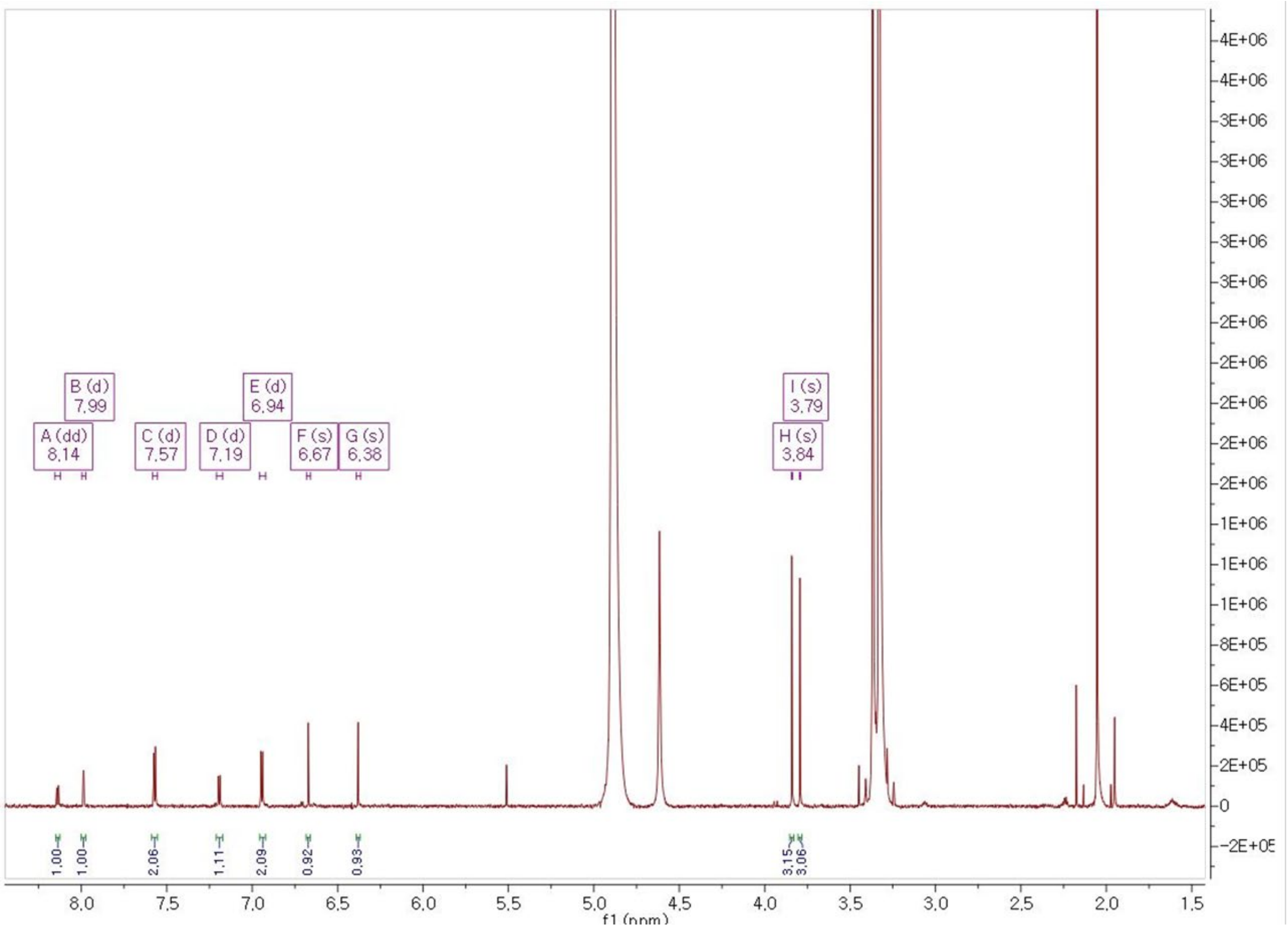

Figure S17. The HPLC-UV chromatographic data for isolation (A), purity verification data (B), UV spectrum (C), and ESI-MS data (D) of **6**

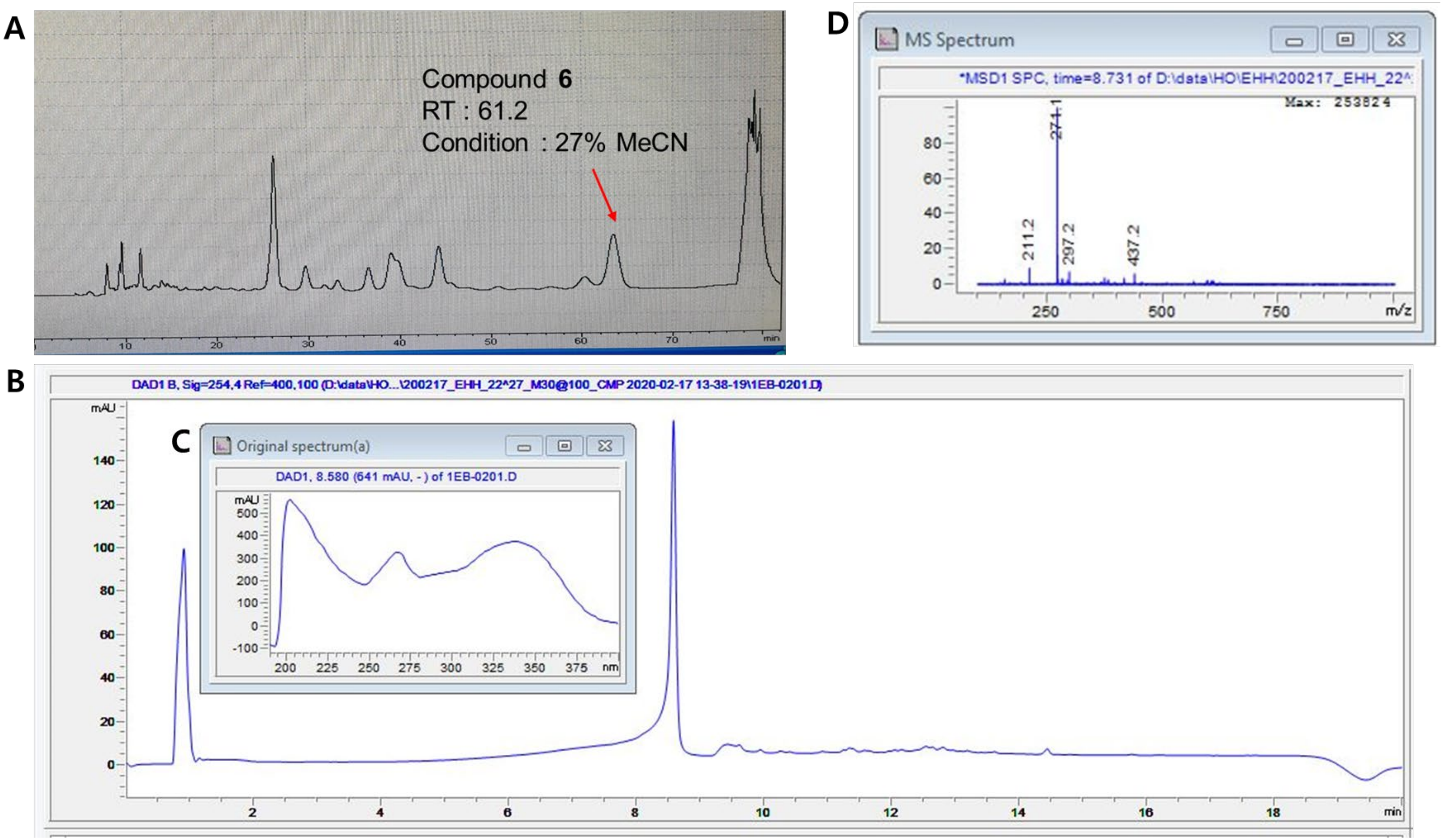

UV (MeOH) λ_{max} (log ε) 205 (4.2), 265 (2.7), 335 (3.2) nm;

1) 205 nm - This high-energy band is typically associated with the $\pi \rightarrow \pi^*$ transitions in the benzene ring. Aromatic rings in flavonoids contribute to this transition due to their conjugated π-electrons. The strong molar absorptivity (log ε) signifies an intense transition, characteristic of electronic excitation within aromatic systems.

2) 265 nm: This band represents another $\pi \rightarrow \pi^*$ transition, involving conjugated double bonds within the flavonoid structure, particularly between the aromatic rings and any unsaturated functional groups. This medium-energy band is indicative of extended conjugation within the molecule, with lower intensity compared to 205 nm due to reduced transition probability.

3) 335 nm: This band corresponds to an $n \rightarrow \pi^*$ transition, typically involving non-bonding electrons on oxygen atoms (such as those in hydroxyl or carbonyl groups) interacting with the conjugated system. This absorption reflects charge transfer within the flavonoid's chromophores, such as interactions between aromatic rings and functional groups like carbonyls. The higher log ε value compared to 265 nm suggests that this transition is relatively more allowed in this case.

Figure S18. The ^{1}H NMR spectrum of **6** (DMSO-d_6, 850 MHz)

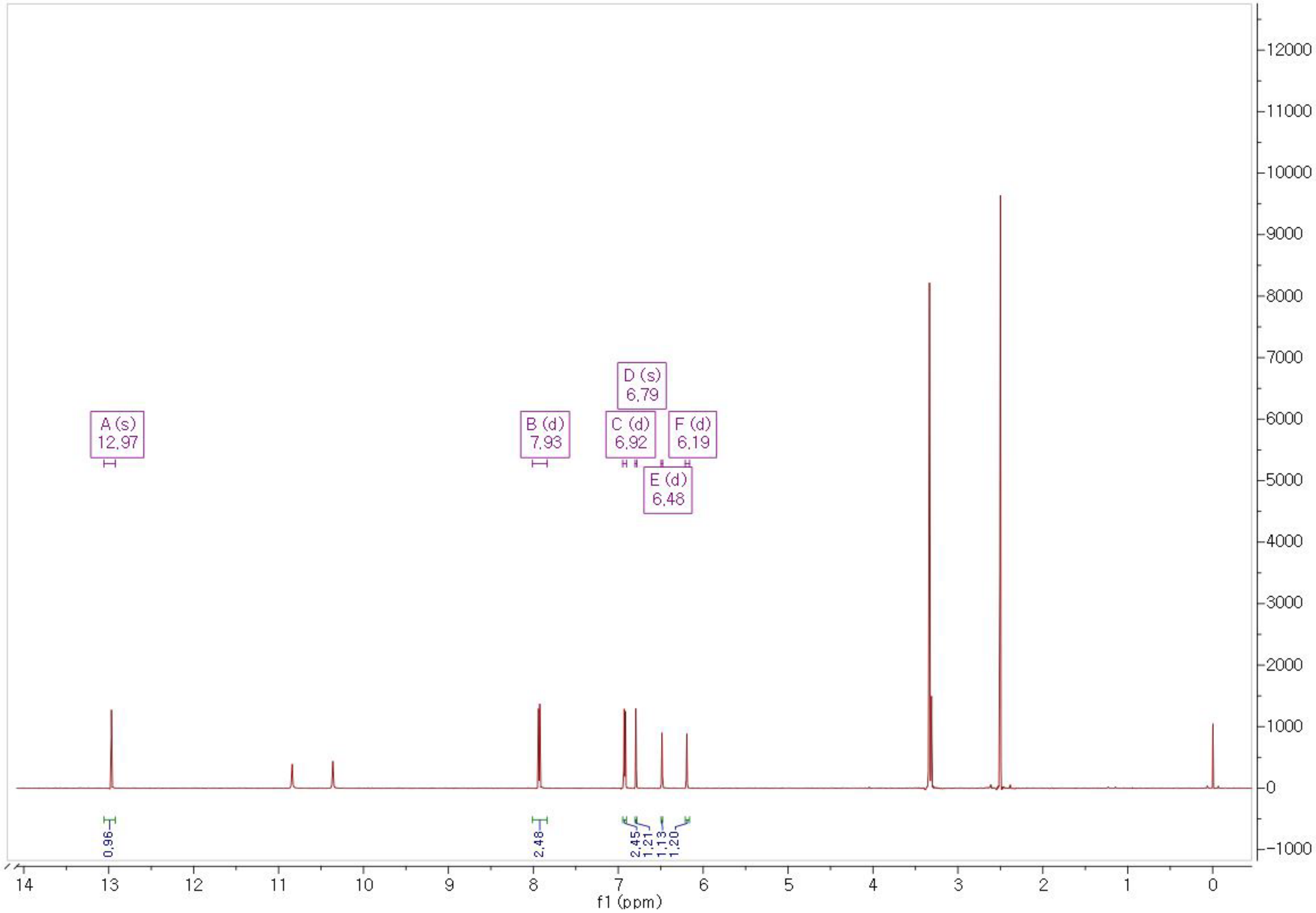

Figure S19. Isolation scheme of compounds from the hexane-soluble fraction

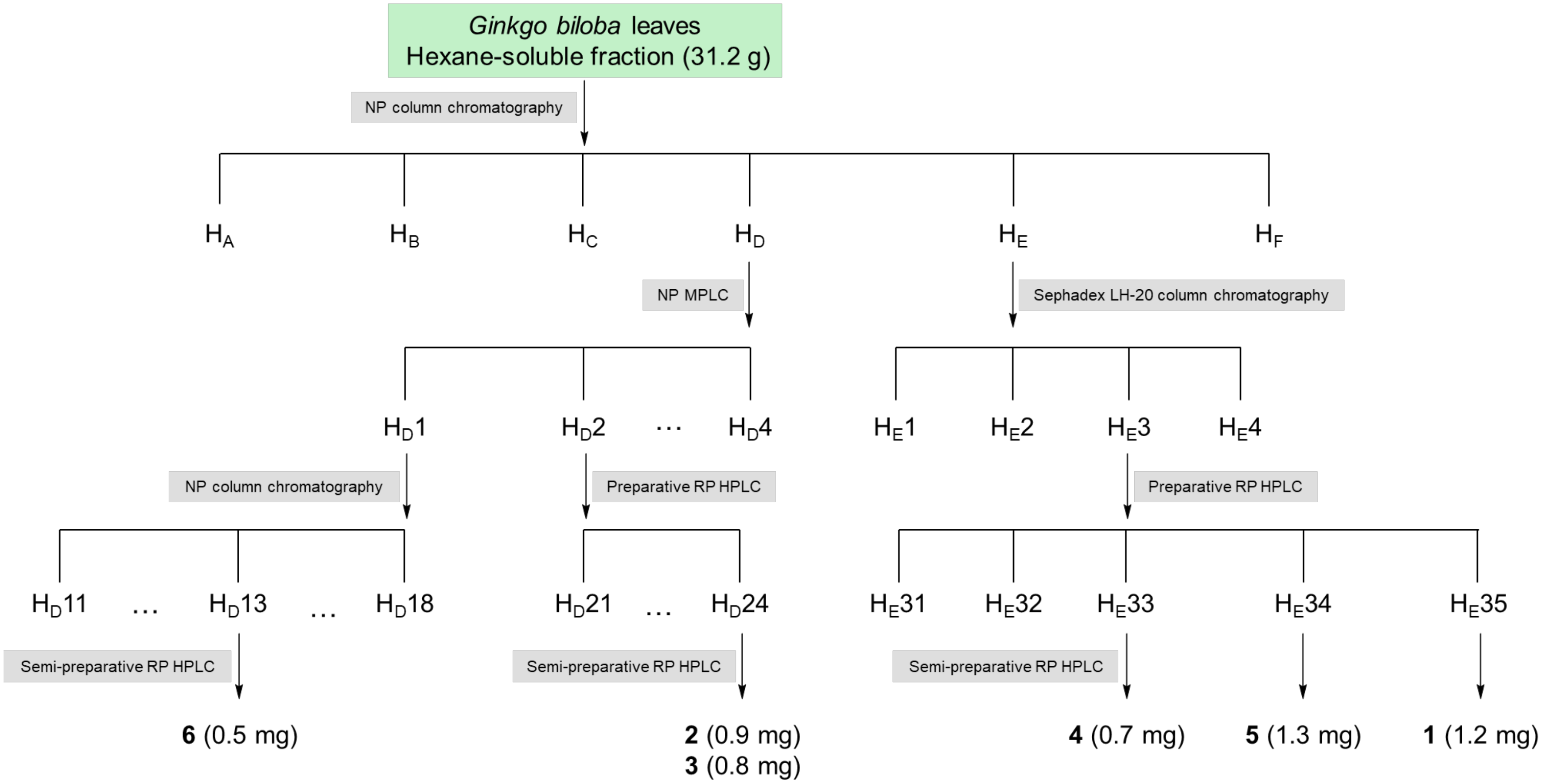

Table S1. The ^{1}H NMR (CD_3OD, 850 MHz) data of compounds **2–5**

Position	**2**	**3**
	δ_H, (J in Hz)	δ_H, (J in Hz)
1		
2		
3	6.69, s	6.69, s
4		
5		
6	6.33, d (2.0)	6.22, d (2.0)
7		
8	6.61, d (2.0)	6.45, d (2.0)
9		
10		
1'		
2'	7.99, d (2.0)	7.98, d (2.0)
3'		
4'		
5'	7.45, d (8.5)	7.34, d (8.5)
6'	8.05, dd (8.5, 2.0)	8.11, dd (8.5, 2.0)
1''		
2''		
3''	6.60, s	6.68, s
4''		
5''		
6''	6.35, s	6.40, s
7''		
8''		
9''		

10''		
1'''		
2'''	7.45, d (9.0)	7.57, d (9.0)
3'''	6.73, d (9.0)	6.91, d (9.0)
4'''		
5'''	6.73, d (9.0)	7.57, d (8.8)
6'''	7.45, d (9.0)	6.91, d (8.8)
7-OCH_3	3.83, s	
4'-OCH_3	3.82, s	3.84, s
4'''-OCH_3		3.80, s

Position	4
	δ_H, (*J* in Hz)
Aglycone	
1	
2	
3	
4	
5	
6	6.18, d (2.0)
7	
8	6.19, d (2.0)
9	
10	
1'	
2'	7.69, d (8.5)
3'	6.93, d (8.5)
4'	
5'	6.93, d (8.5)
6'	7.69, d (8.5)
Rhamnose	
1''	5.83, s
2''	4.40, d (3.5)
3''	3.84, dd (9.5, 3.5)
4''	3.44, t (9.5)
5''	3.57, dd (9.5, 6.5)
6''	1.07, d (6.5)
Glucose	

1'''	4.49, d (8.0)
2'''	3.30, m[b]
3'''	3.30, m[b]
4'''	3.38, t (10.0)
5'''	3.52, ddd (10.0, 7.0, 2.5)
6'''	4.13, dd (12.0, 7.0); 4.58, dd (12.0, 2.5)
Coumaroyl	
1''''	
2''''	7.21, d (8.5)
3''''	6.69, d (8.5)
4''''	
5''''	6.69, d (8.5)
6''''	7.21, d (8.5)
7''''	7.43, d (16.0)
8''''	6.01, d (16.0)
9''''	

Position	**5**
	δ_H, (*J* in Hz)
1	
2	
3	6.67, s
4	
5	
6	6.38, s
7	
8	
9	
10	
1'	
2'	7.57, d (8.5)
3'	6.94, d (8.5)
4'	
5'	6.94, d (8.5)
6'	7.57, d (8.5)
1''	
2''	7.99, d (2.0)
3''	
4''	
5''	7.19, d (8.5)
6''	8.14, dd (8.5, 2.0)
4'-OCH_3	3.84, s
4''-OCH_3	3.79, s

Table S2. The ^{1}H NMR (DMSO-d_6, 850 MHz) data of compound **6**

Position	**6** δ_H, (J in Hz)
1	
2	
3	6.79, s
4	
5	
6	6.19, d (2.0)
7	
8	6.48, d (2.0)
9	
10	
1'	
2'	7.93, d (8.5)
3'	6.92, d (8.5)
4'	
5'	6.92, d (8.5)
6'	7.93, d (8.5)
5-OH	12.97, s
